# Supplementary material for: Chemoproteomics identifies protein ligands for monoacylglycerol lipids
Source: Commun Chem. 2025 Jul 4;8:197. doi: 10.1038/s42004-025-01589-w (PMC12227648; doi:10.1038/s42004-025-01589-w)
Supplement: Supplementary file 1 — Supplementary Information [file 42004_2025_1589_MOESM1_ESM.pdf]

SUPPLEMENTARY INFORMATION

Chemoproteomics Identifies Protein Ligands for Monoacylglycerol Lipids

Karthik Shanbhag[1,4], Amol B. Mhetre[1,4,5], Ojal Saharan[1], Archit Devarajan[1,6], Anisha Rai[1,7], M. S. Madhusudhan[1,2], Harinath Chakrapani[3,*], Siddhesh S. Kamat[1,8,*]

[1]Department of Biology, Indian Institute of Science Education and Research, Dr. Homi Bhabha Road, Pashan, Pune 411008, Maharashtra, India.

[2]Department of Data Science, Indian Institute of Science Education and Research, Dr. Homi Bhabha Road, Pashan, Pune 411008, Maharashtra, India.

[3]Department of Chemistry, Indian Institute of Science Education and Research, Dr. Homi Bhabha Road, Pashan, Pune 411008, Maharashtra, India.

[4]These authors contributed equally

[5]Present address: Department of Pathology, University of Michigan Medical School, Ann Arbor 48109, Michigan, USA.

[6]Department of Biology, New York University, New York 10003, New York, USA, and New York Genome Center, New York 10013, New York, USA.

[7]Present address: Department of Chemistry and Biochemistry, Georgia Institute of Technology, North Avenue, Atlanta 30332, Georgia, USA.

[8]Lead Contact

*To whom the correspondence can be made: siddhesh@iiserpune.ac.in; harinath@iiserpune.ac.in

SUPPLEMENTARY INFORMATION

TABLE OF CONTENTS

Supplementary Figures 1 – 20 3 – 22

Supplementary Data 1 – 4 23 – 26

- See also separate excel sheets

Supplementary Synthetic Note 27 – 46

Supplementary References 47 – 48

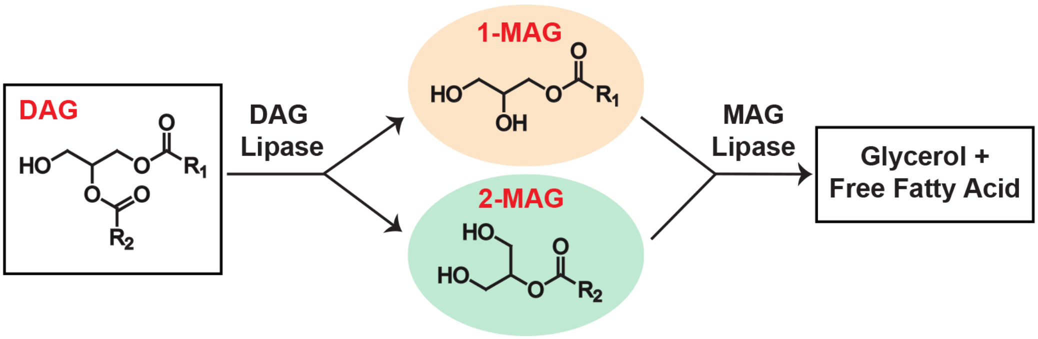

Supplementary Figure 1. Metabolism of MAG lipids. Monoacylglycerol (1-MAG and 2-MAG) lipids are biosynthesized from diacylglycerol (DAG) precursors by the action of dedicated DAG lipases. On the other hand, they are degraded by the action of MAG lipases to form glycerol and free fatty acids.

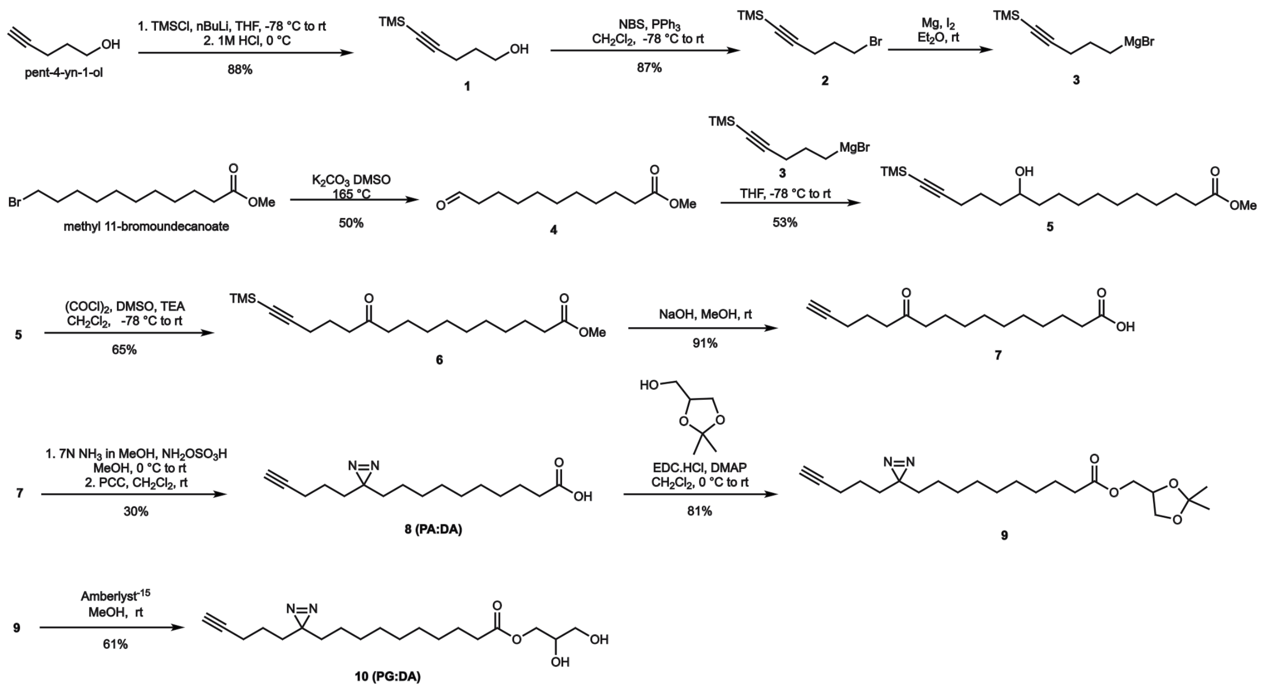

Supplementary Figure 2. The synthetic scheme used to generate the PG-DA probe. Complete details of all the synthesis and analytical characterization of the various intermediates can be found in the **Supplementary Synthetic Note**.

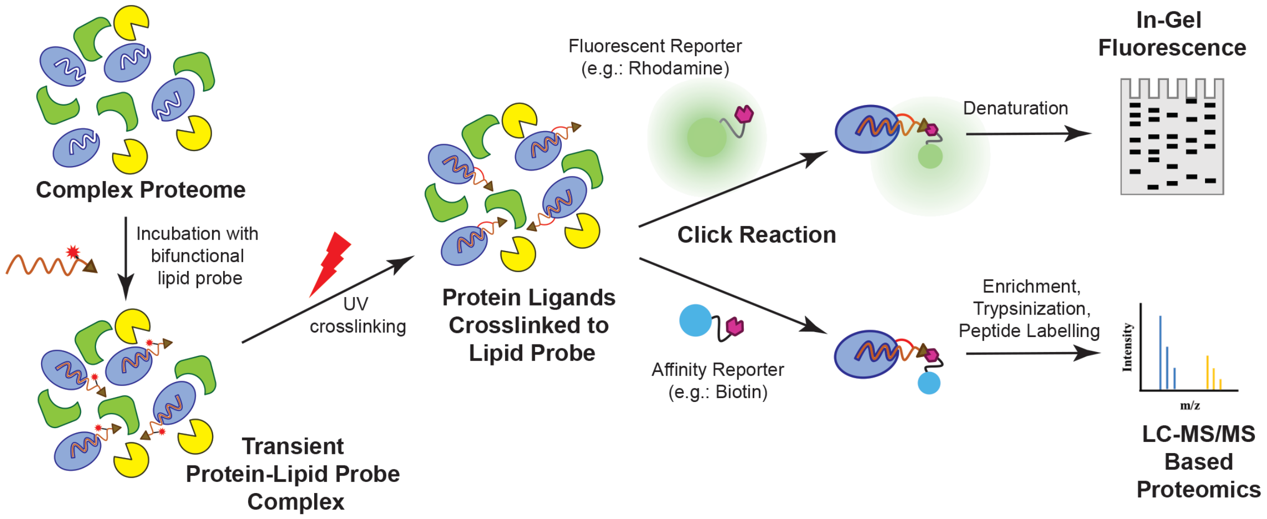

Supplementary Figure 3. The photoaffinity labeling strategy. A general workflow for the photoaffinity labeling strategy using both the in-gel fluorescence and the LC-MS/MS based proteomics platforms used in this study.

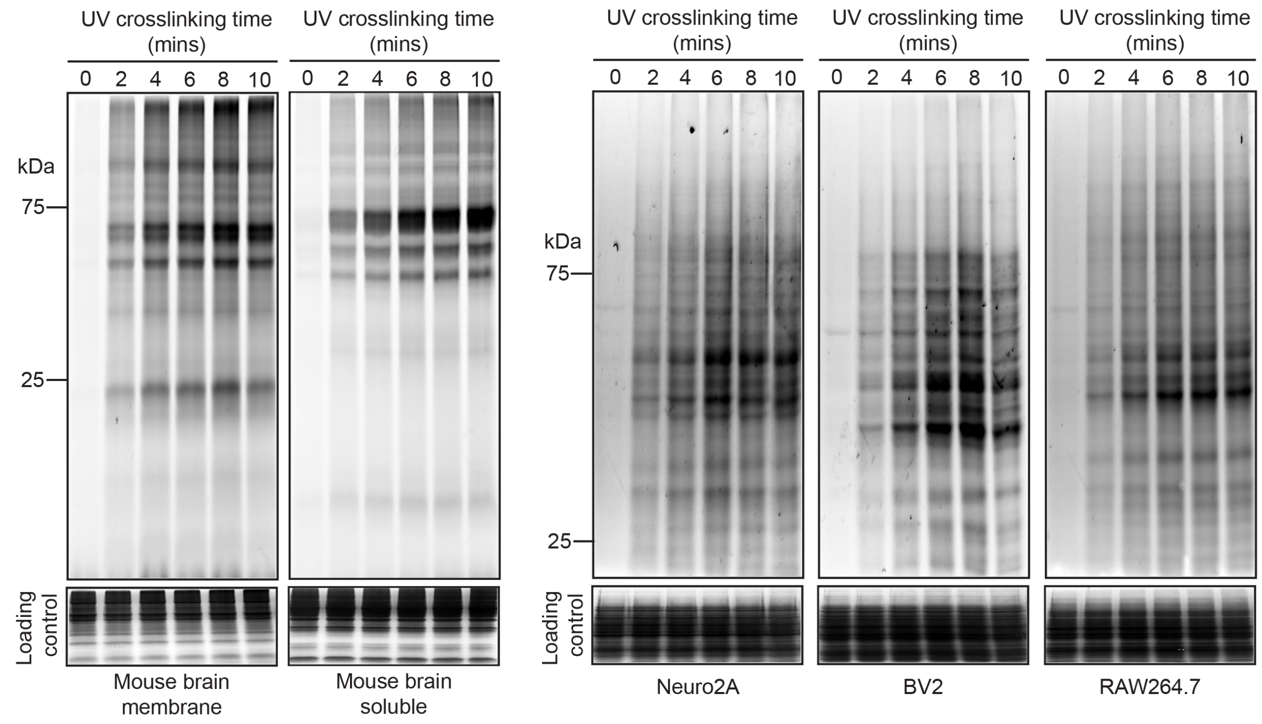

Supplementary Figure 4. UV-dependent crosslinking of the PG-DA probe (500 μM) in various lysates. In this experiment, UV-crosslinking time was varied from 0 – 10 mins, and in all cases, 6 mins was found to be the optimal time for UV crosslinking. The Coomassie staining shows the loading control for all gels in this experiment. This experiment was done three times with reproducible results each time.

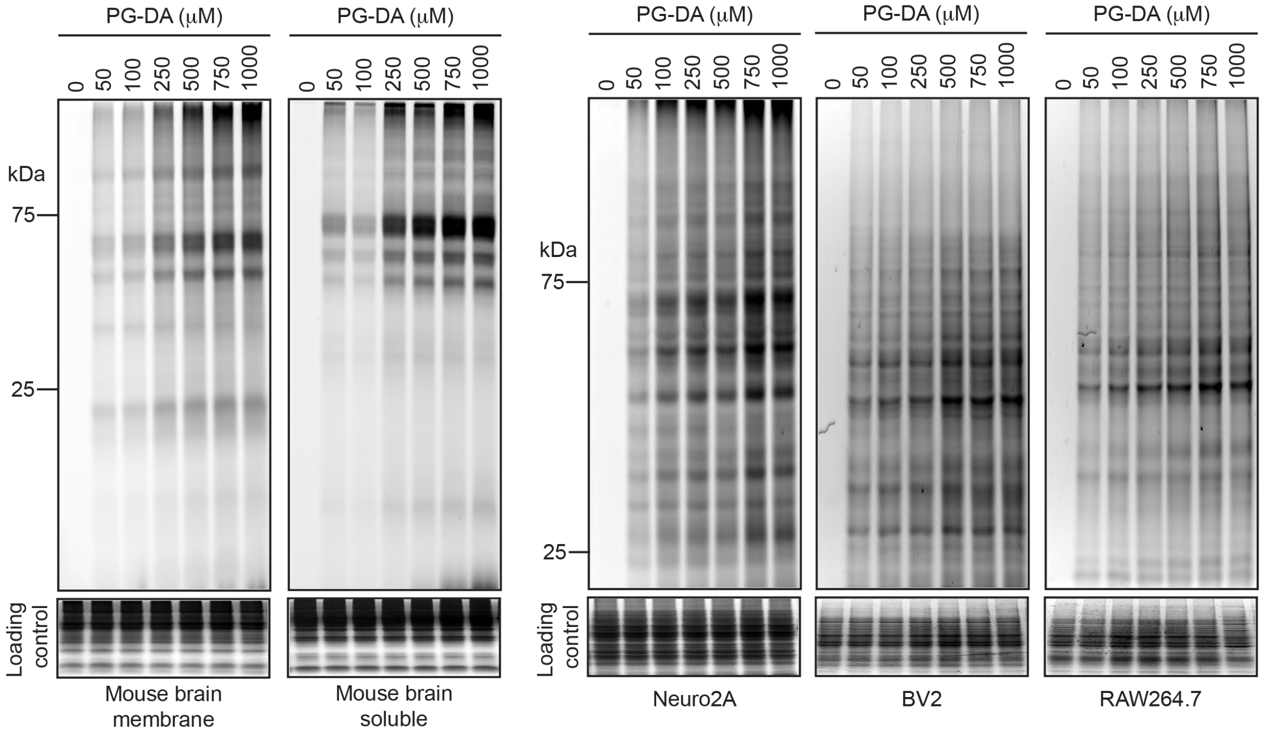

Supplementary Figure 5. UV-dependent crosslinking of various concentrations of the PG-DA probe (0 – 1000 μM) in various lysates. In this experiment, UV-crosslinking time was kept constant at 10 mins. The Coomassie staining shows the loading control for all gels in this experiment. This experiment was done three times with reproducible results each time.

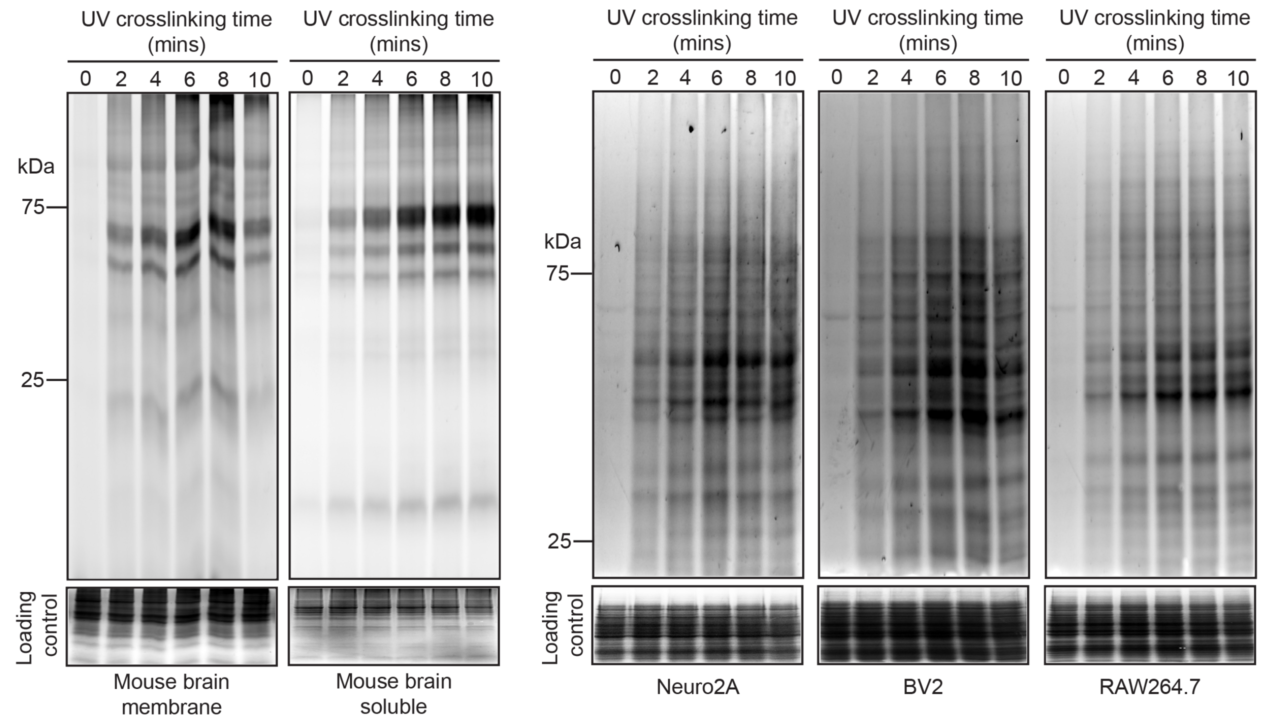

Supplementary Figure 6. UV-dependent crosslinking of the PA-DA probe (500 μM) in various lysates. In this experiment, UV-crosslinking time was varied from 0 – 10 mins, and in all cases, 6 mins was found to be the optimal time for UV crosslinking. The Coomassie staining shows the loading control for all gels in this experiment. This experiment was done three times with reproducible results each time.

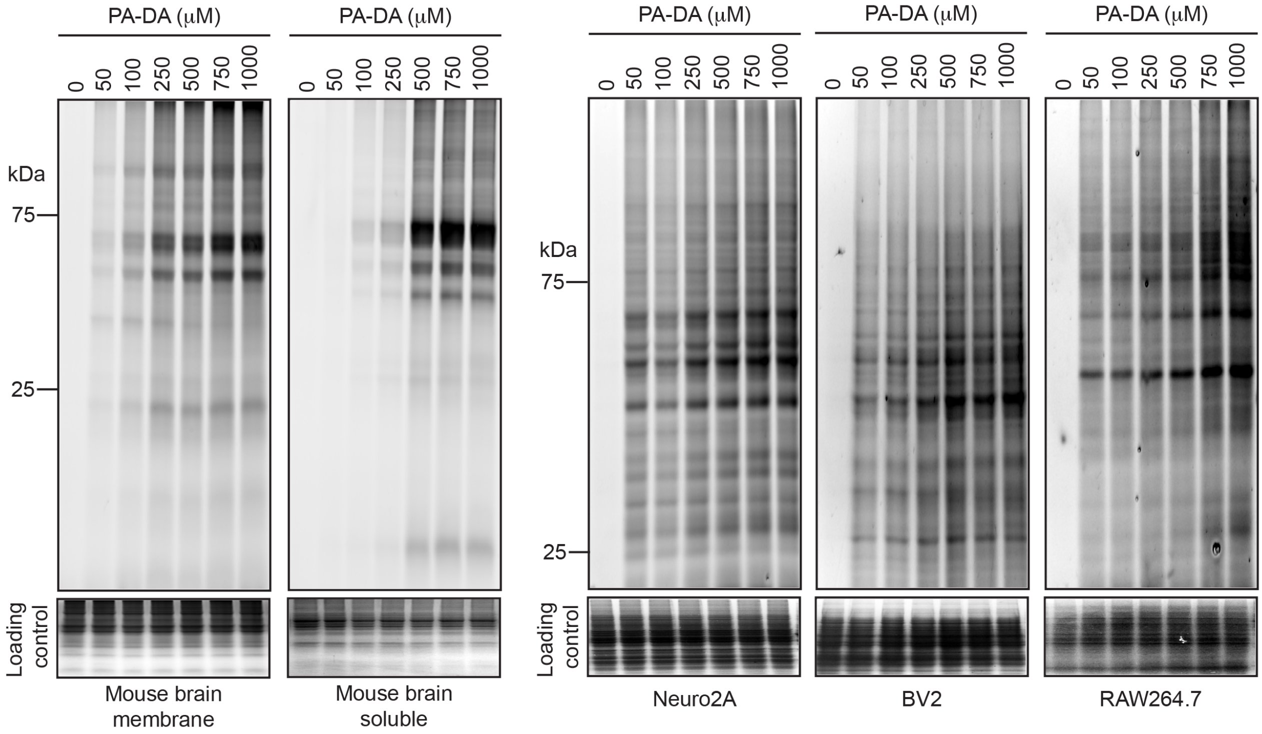

Supplementary Figure 7. UV-dependent crosslinking of various concentrations of the PA-DA probe (0 – 1000 μM) in various lysates. In this experiment, UV-crosslinking time was kept constant at 10 mins. The Coomassie staining shows the loading control for all gels in this experiment. This experiment was done three times with reproducible results each time.

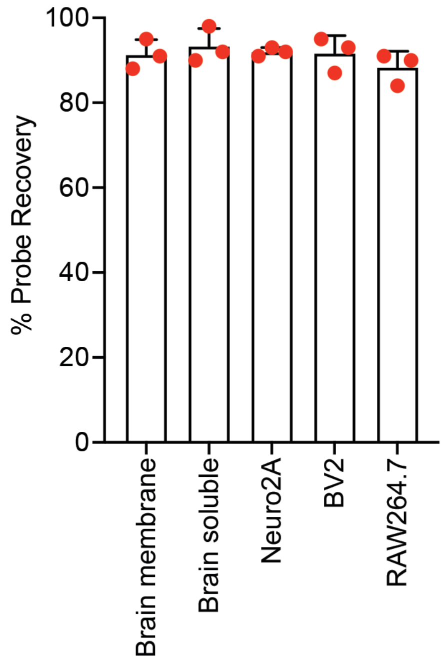

Supplementary Figure 8. Recovery of the PG-DA probe from various lysates. 50 nmol of the PG-DA probe was added to 1 mg of various lysates, and allowed to incubate under our experimental conditions (30 mins at 37 °C, 2 mM PMSF added for PG-DA experiments). Thereafter, the lysates were extracted using protocols reported previously[1-4], to enrich the intact PG-DA probe, and the molar concentration was estimated using LC-MS analysis previously reported by us[3,4]. The PG-DA probe was assessed in the positive ion mode as the $(M+NH_4)^+$ species. For the PG-DA probe, under our experimental conditions, we found that a significant amount of the probe was intact (not degraded) in all the lysates.

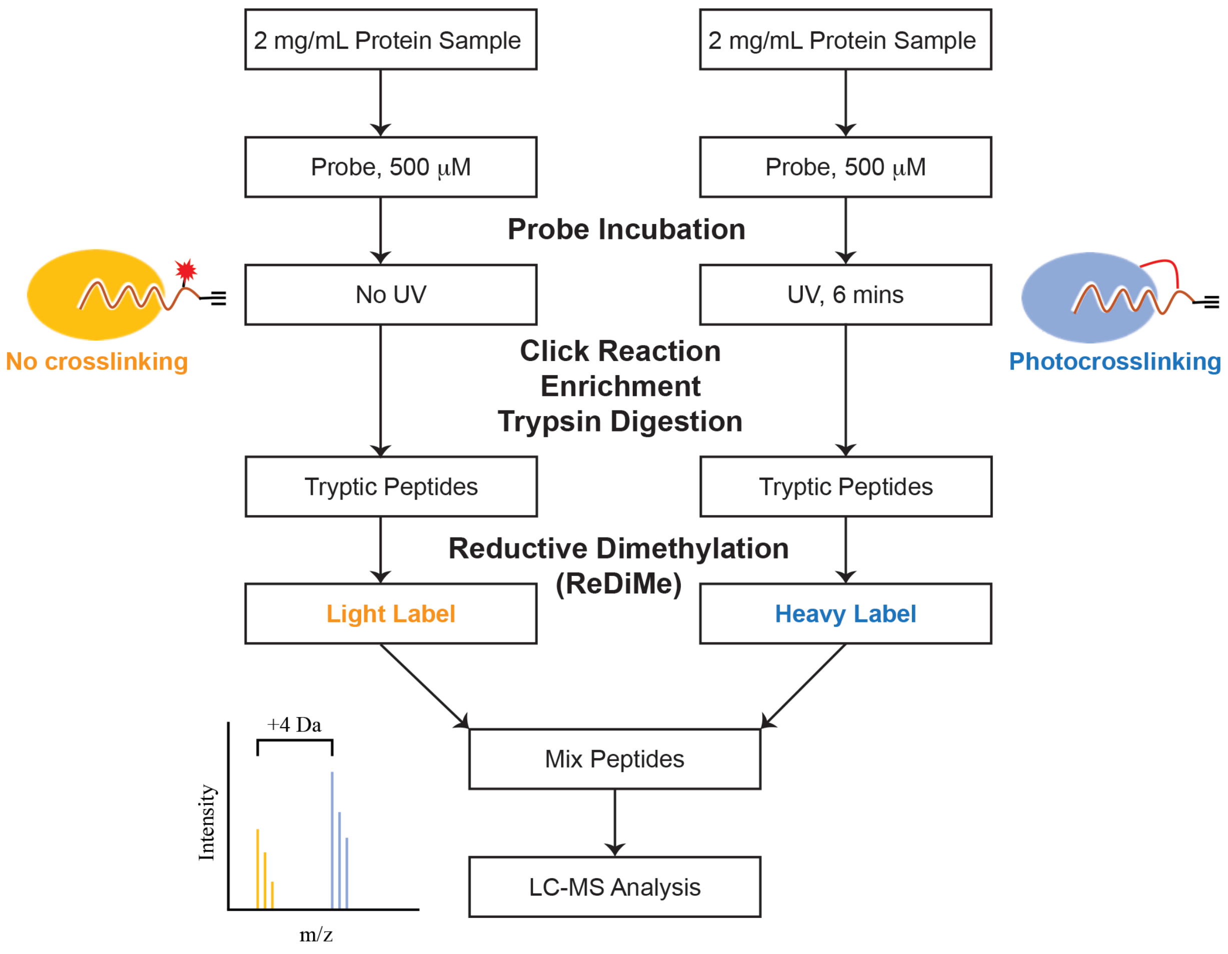

Supplementary Figure 9. A general workflow of the LC-MS/MS based quantitative chemoproteomics experiment for identifying total set of protein enriched by either the PG-DA or PA-DA probe in a UV-dependent manner from various mammalian lysates.

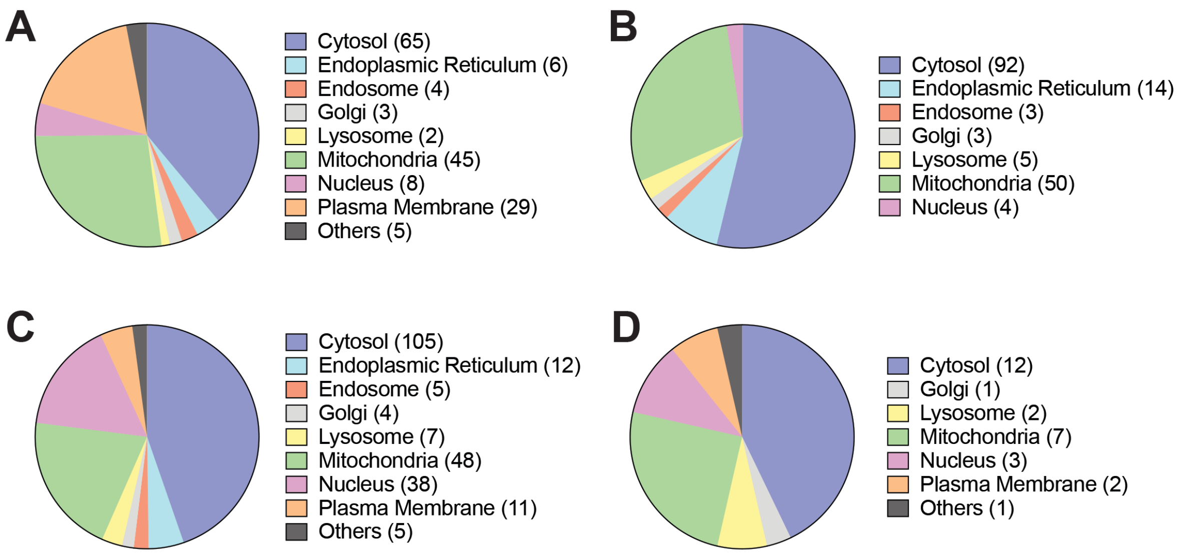

Supplementary Figure 10. Cellular localization of the proteins enriched by the PG-DA probe from the lysates of: (**A**) mouse brain, (**B**) Neuro2A cells, (**C**) RAW264.7 cells, and (**D**) BV2 cells, as per the UniProt database[5].

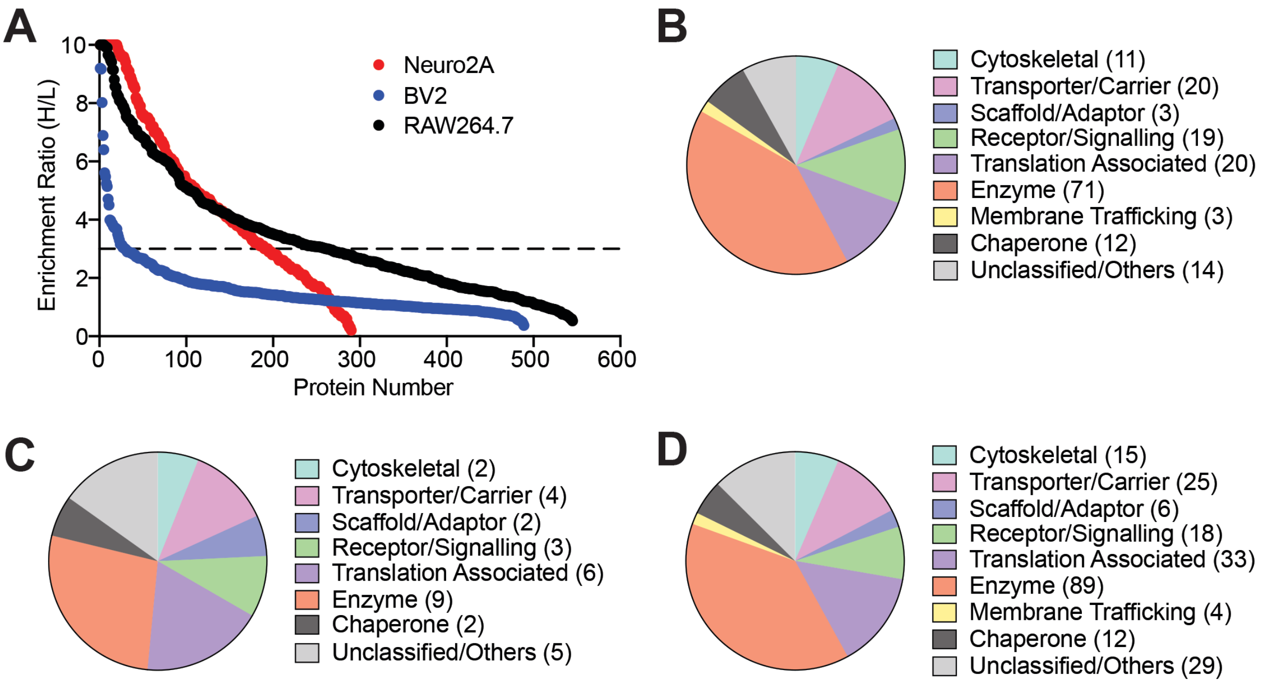

Supplementary Figure 11. (**A**) A LC-MS/MS based chemical proteomics experiment showing enrichment ratio (heavy:light; H:L) of the total proteins identified from the UV-dependent photocrosslinking of the PG-DA probe (500 μM, 6 mins of UV exposure) from the lysates of various immortalized mammalian cell lines. Each data point represents the mean of the enrichment ratio obtained for the respective protein from two or three biological replicate for a particular proteomic fraction, based on the defined filtering criteria for this proteomics experiment. The horizontal dotted line denotes an enrichment ratio ≥ 3, and proteins having an enrichment ratio above this threshold were considered enriched by the PG-DA probe, and taken forward for subsequent analysis. Complete details for all the proteins can be found in **Supplementary Data 1**. (**B–D**) Categorization of protein classes enriched by the PG-DA probe based on the Panther database annotation[6,7] for: (**B**) Neuro2A; (**C**) BV2; and (**D**) RAW264.7 cells respectively.

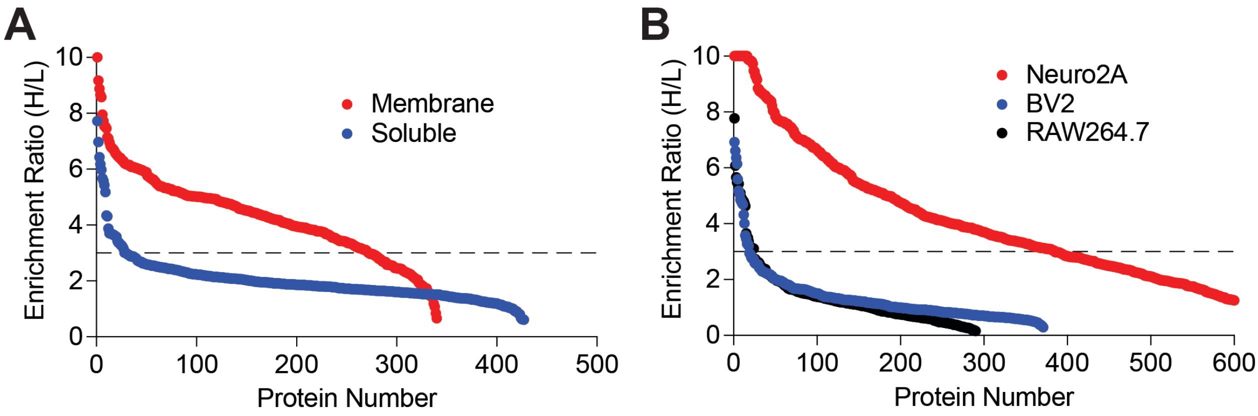

Supplementary Figure 12. A LC-MS/MS based chemical proteomics experiment showing enrichment ratio (heavy:light; H:L) of the total proteins identified from the UV-dependent photocrosslinking of the PA-DA probe (500 μM, 6 mins of UV exposure) from (**A**) the membrane and soluble lysates prepared from the mouse brain, and (**B**) from lysates of various immortalized mammalian cell lines. Each data point represents the mean of the enrichment ratio obtained for the respective protein from two or three biological replicate for a particular proteomic fraction, based on the defined filtering criteria for this proteomics experiment. The horizontal dotted line denotes an enrichment ratio ≥ 3, and proteins having an enrichment ratio above this threshold were considered enriched by the PA-DA probe, and taken forward for subsequent analysis. Complete details for all the proteins can be found in **Supplementary Data 2**.

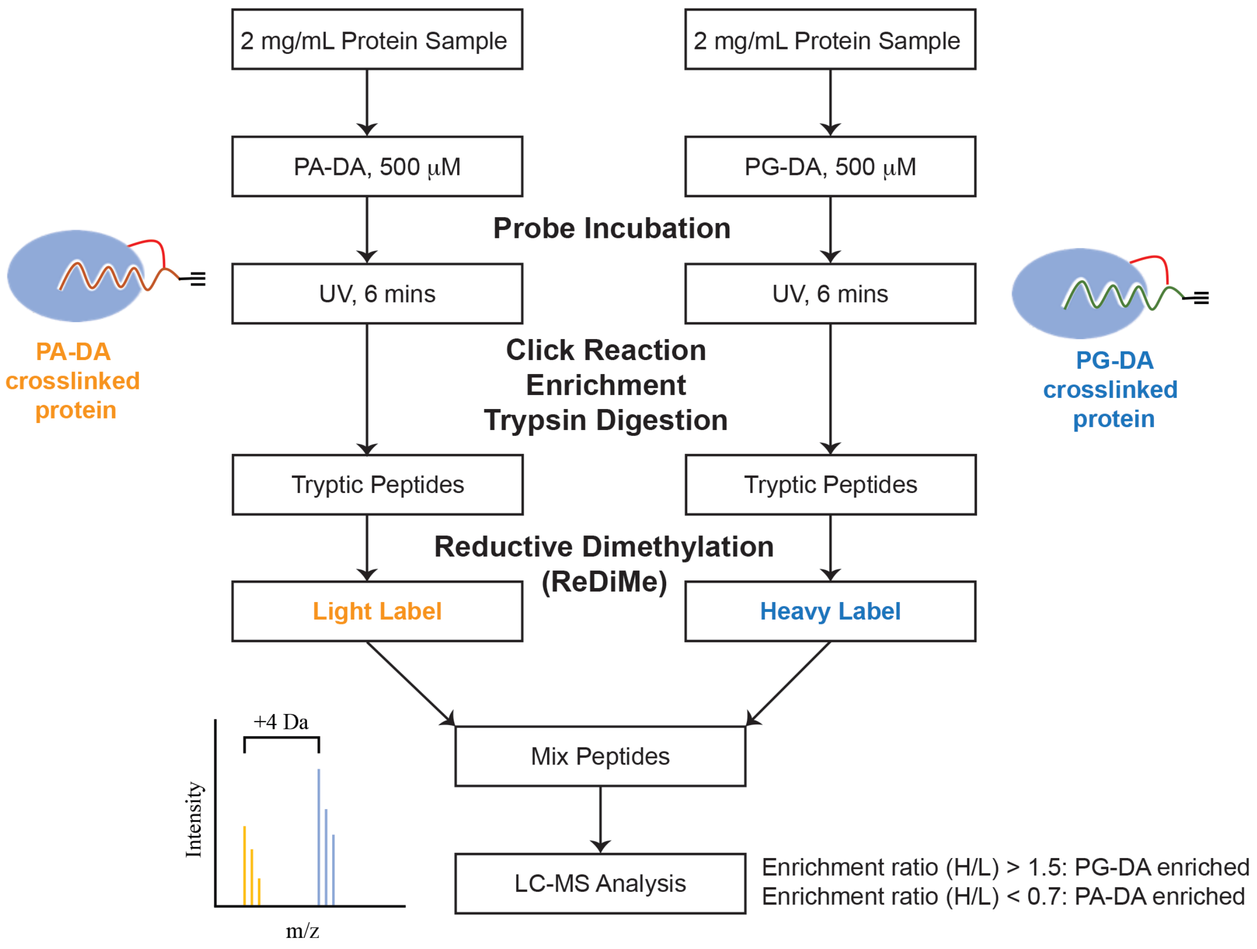

Supplementary Figure 13. A general workflow of the competitive LC-MS/MS based quantitative chemoproteomics experiment for identifying total set of protein enriched by either the PG-DA or PA-DA probe in a UV-dependent manner from various mammalian lysates.

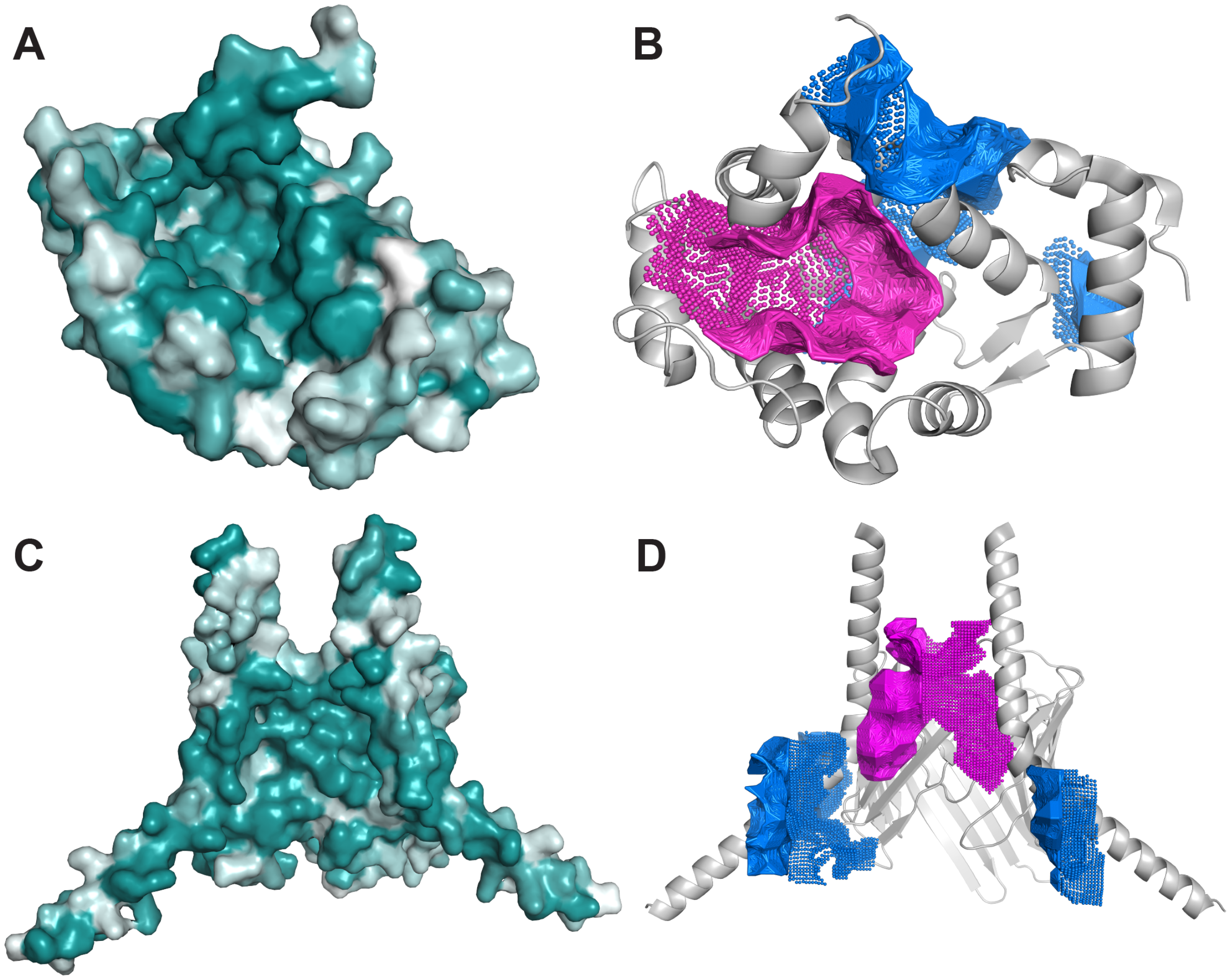

Supplementary Figure 14. Identification of potential hydrophobic 1-PG binding cavities in HPCA and TOMM22. (**A, B**) 1-PG binding cavities identified in HPCA using the (**A**) DEPTH[8], and (**B**) CavityPlus web servers[9]. (**C, D**) 1-PG binding cavities identified in TOMM22 using the (**C**) DEPTH[8], and (**D**) CavityPlus web servers[9].

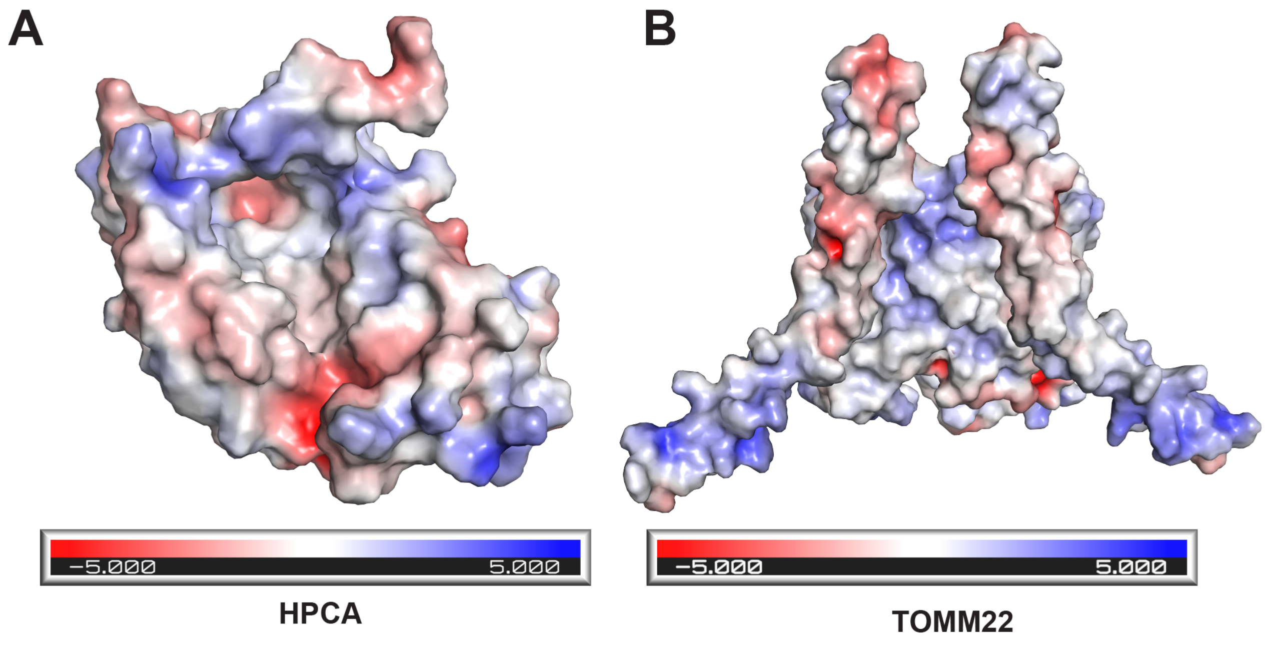

Supplementary Figure 15. Electrostatic surface map of (**A**) HPCA and (**B**) TOMM22, showing charges and hydrophobic pockets.

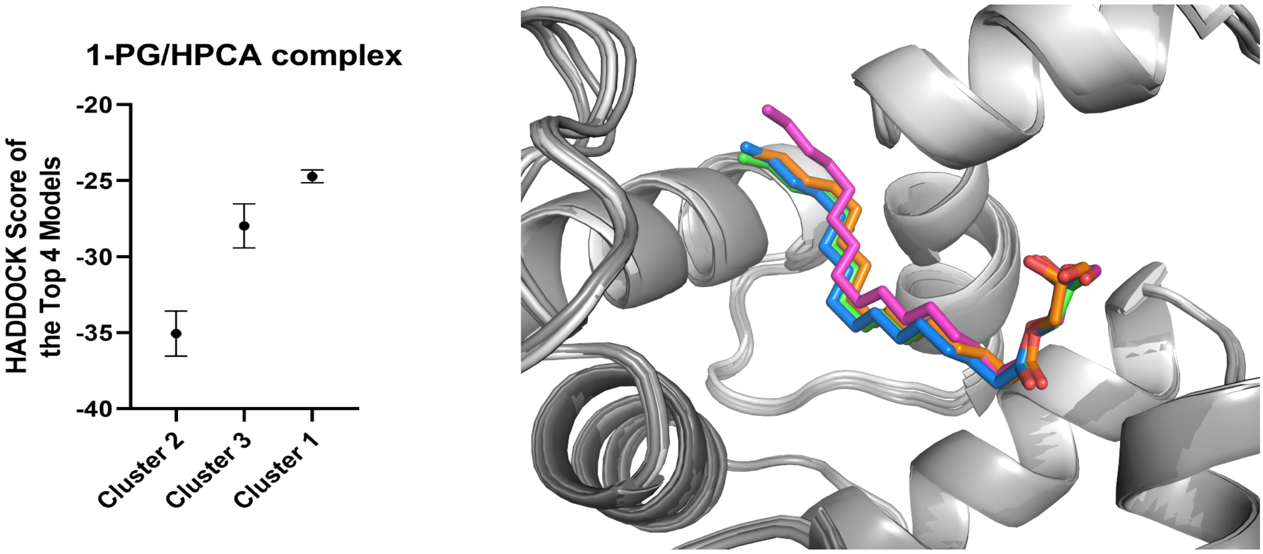

Supplementary Figure 16. (*Left*) The average HADDOCK score of the top four 1-PG/HPCA models in each cluster generate by HADDOCK[10]. (*Right*) Overlay of the top four 1-PG/HPCA models from the best cluster (cluster 2) showing ligand orientation in the binding pocket.

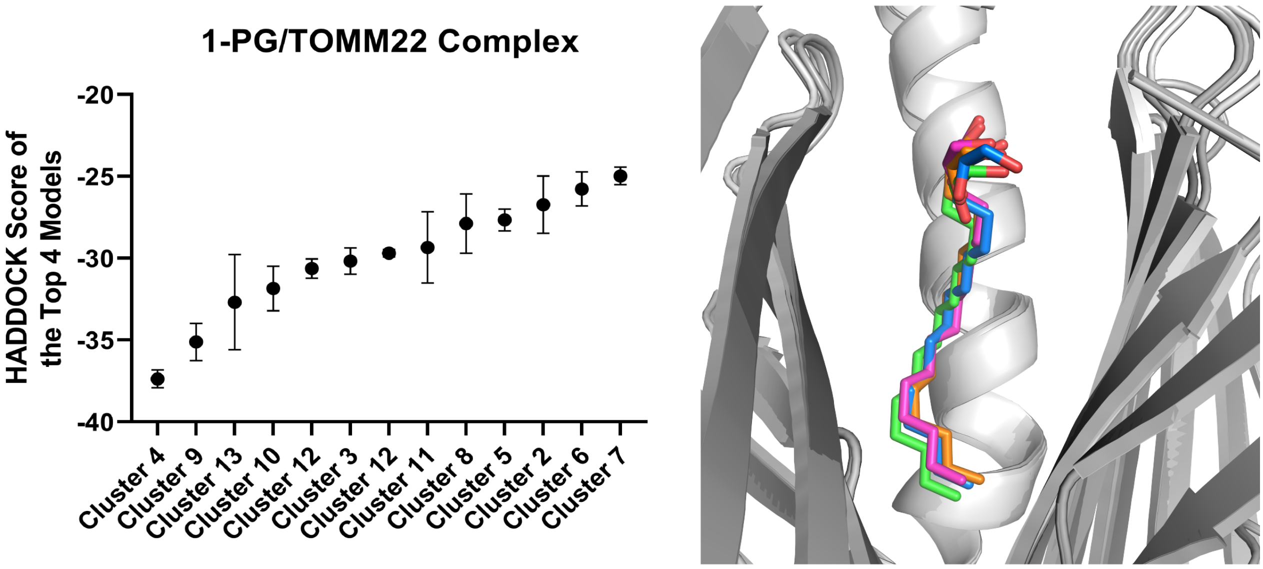

Supplementary Figure 17. (*Left*) The average HADDOCK score of the top four 1-PG/TOMM22 models in each cluster generate by HADDOCK[10]. (*Right*) Overlay of the top four 1-PG/TOMM22 models from the best cluster (cluster 9) showing ligand orientation in the binding pocket.

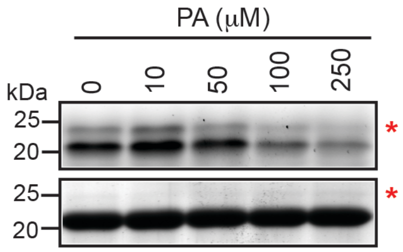

Supplementary Figure 18. The effect of incubating increasing concentrations of palmitic acid (0 – 250 μM) on the binding of the PA-DA probe (10 μM) to recombinantly purified mouse HPCA (10 μM). The top panel shows the in-gel fluorescence, while the bottom panel is the Coomassie staining as the loading control for this respective gel. The red asterisk denotes the minor form of HPCA at 25-kDa in both gels. All other experimental conditions remain similar to those reported in Figure 6, and this particular experiment was done three times with reproducible results each time.

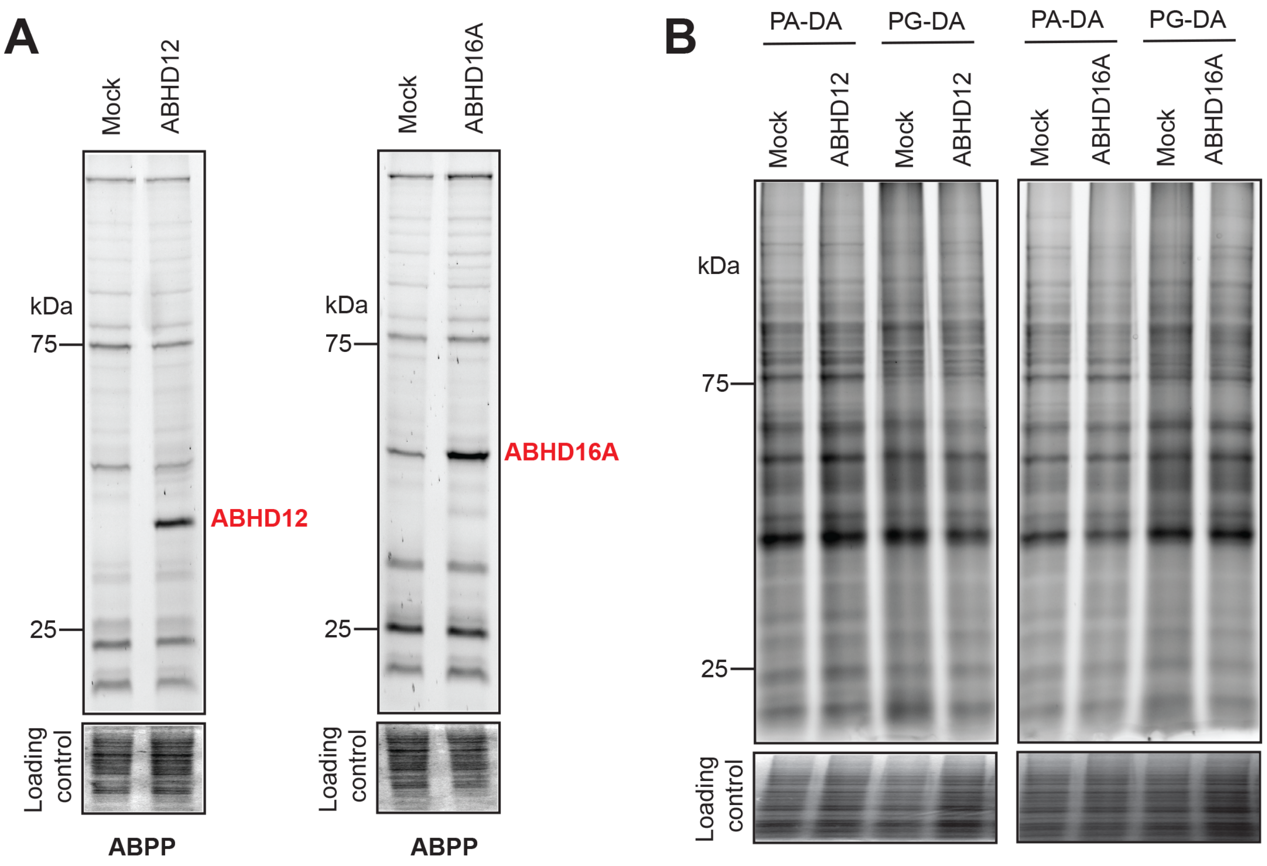

Supplementary Figure 19. (**A**) Representative activity-based protein profiling (ABPP)[2,11,12] gels confirming the overexpression of ABHD12 and ABHD16A in cellular lysates of HEK293T cells relative to a mock transfection control. In this experiment, 100 μg lysates were used per condition (100 μL of 1 mg/mL), and the ABPP probe, FP-rhodamine was used at a final concentration of 2 μM. The Coomassie staining shows the loading control for all gels in this experiment. This experiment was done two times with reproducible results each time. (B) A representative in-gel fluorescence experiment showing the no binding of the PG-DA or PA-DA probe, to either ABHD12 or ABHD16A in HEK293T cellular lysates overexpressing these lipases. In this experiment, 200 μg lysates were used per condition (100 μL of 2 mg/mL), and the probes were used at 50 μM final concentration, similar to the conditions in Figure 7A. The Coomassie staining shows the loading control for all gels in this experiment. This experiment was done three times with reproducible results each time.

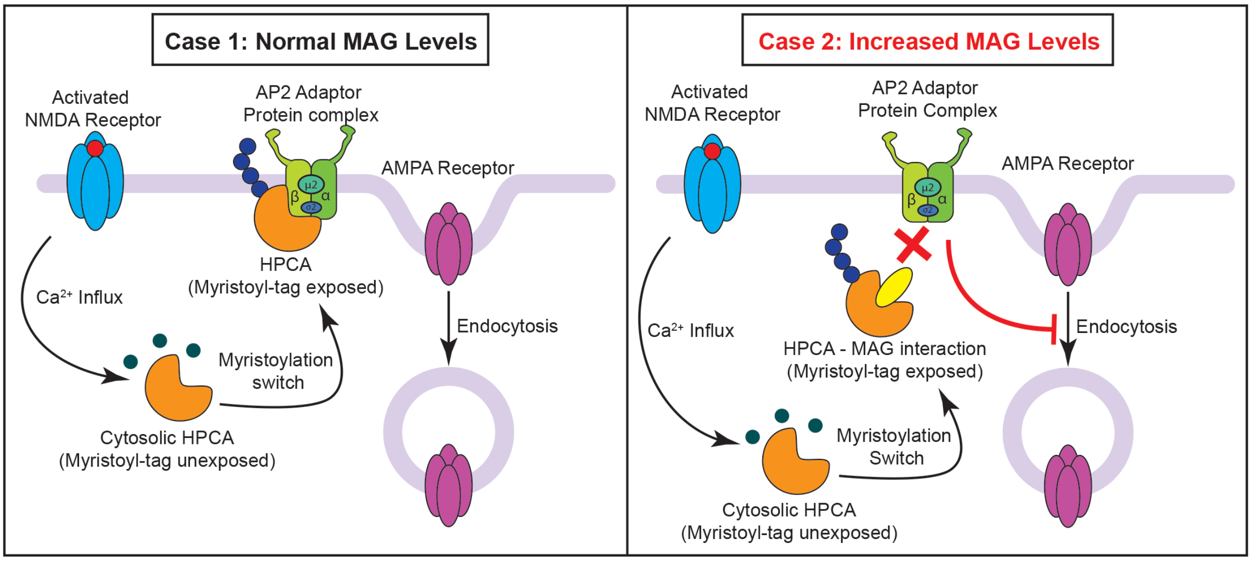

Supplementary Figure 20. A possible model for MAG's effect on the HPCA-calcium sensing. See *Discussion* section for details.

SUPPLEMENTARY DATA 1: A LC-MS/MS based chemoproteomic characterization of the PG-DA probe as a function of UV-crosslinking in different mammalian lysates.

In all cases of these chemical proteomics experiments involving post-tryptic reductive demethylation (ReDiMe)[2], the tryptic peptides from the UV-crosslinked sample was labelled with heavy formaldehyde, while the tryptic peptides from the no UV sample was labelled with light formaldehyde. An enrichment ratio (H/L) represent relative abundance of a peptide in the heavy labelled sample versus the light labelled sample. For a protein to be classified as enriched by the PG-DA probe upon UV-crosslinking, it needed to be identified in at least 2 out of 3 replicates, have $\geq$ 3 quantified peptides per replicate, and have an enrichment ratio $\geq$ 3 in all replicates.

Tab A: List of proteins enriched by the PG-DA probe in the mouse brain membrane lysates.
Tab B: List of proteins enriched by the PG-DA probe in the mouse brain soluble lysates.
Tab C: List of proteins enriched by the PG-DA probe in the lysates obtained from Neuro2A cells.
Tab D: List of proteins enriched by the PG-DA probe in the lysates obtained from RAW264.7 cells.
Tab E: List of proteins enriched by the PG-DA probe in the lysates obtained from BV2 cells.

SUPPLEMENTARY DATA 2: A LC-MS/MS based chemoproteomic characterization of the PA-DA probe as a function of UV-crosslinking in different mammalian lysates.

In all cases of these chemical proteomics experiments involving post-tryptic reductive demethylation (ReDiMe)[2], the tryptic peptides from the UV-crosslinked sample was labelled with heavy formaldehyde, while the tryptic peptides from the no UV sample was labelled with light formaldehyde. An enrichment ratio (H/L) represent relative abundance of a peptide in the heavy labelled sample versus the light labelled sample. For a protein to be classified as enriched by the PA-DA probe upon UV-crosslinking, it needed to be identified in at least 2 out of 3 replicates, have $\geq$ 3 quantified peptides per replicate, and have an enrichment ratio $\geq$ 3 in all replicates.

<u>**Tab A**</u>: List of proteins enriched by the PA-DA probe in the mouse brain membrane lysates.

<u>**Tab B**</u>: List of proteins enriched by the PA-DA probe in the mouse brain soluble lysates.

<u>**Tab C**</u>: List of proteins enriched by the PA-DA probe in the lysates obtained from Neuro2A cells.

<u>**Tab D**</u>: List of proteins enriched by the PA-DA probe in the lysates obtained from RAW264.7 cells.

<u>**Tab E**</u>: List of proteins enriched by the PA-DA probe in the lysates obtained from BV2 cells.

SUPPLEMENTARY DATA 3: A competitive LC-MS/MS based chemoproteomics experiments comparing the protein ligands of the PG-DA probe versus the PA-DA probe in different mammalian lysates.

In all cases of these chemical proteomics experiments involving post-tryptic reductive demethylation (ReDiMe)[2], the tryptic peptides from the PG-DA treated sample was labelled with heavy formaldehyde, while the tryptic peptides from the PA-DA treated sample was labelled with light formaldehyde. An enrichment ratio (H/L) represent relative abundance of a peptide in the heavy labelled sample versus the light labelled sample. For a protein to be considered for any analysis in this experiment, it needed to be identified in at least 2 out of 3 replicates, and have ≥ 3 quantified peptides per replicate. A protein was considered enriched by the PG-DA probe, if it had an enrichment ratio ≥ 1.5 in all the replicates it was identified, while an enrichment ratio ≤ 0.7 classified a protein to be enriched by the PA-DA probe.

Tab A: Complete list of proteins identified in this competitive probe versus probe (PG-DA vs PA-DA) chemical proteomics experiments performed in the mouse brain membrane lysates.

Tab B: Complete list of proteins identified in this competitive probe versus probe (PG-DA vs PA-DA) chemical proteomics experiments performed in the mouse brain soluble lysates.

Tab C: Complete list of proteins identified in this competitive probe versus probe (PG-DA vs PA-DA) chemical proteomics experiments performed in the lysates from Neuro2A cells.

Tab D: Complete list of proteins identified in this competitive probe versus probe (PG-DA vs PA-DA) chemical proteomics experiments performed in the lysates from BV2 cells.

Tab E: Complete list of proteins identified in this competitive probe versus probe (PG-DA vs PA-DA) chemical proteomics experiments performed in the lysates from RAW264.7 cells.

SUPPLEMENTARY DATA 4. Datasets from the molecular docking of 1-PG into HPCA and TOMM22.

Tab A. Identification of the top 5 cavities by CavityPlus based on the DrugScore for HPCA and TOMM22.

Tab B. Interaction energies for the 1-PG/HPCA and 1-PG/TOMM22 complexes for the top 4 models within the selected clusters.

SUPPLEMENTARY SYNTHETIC NOTE

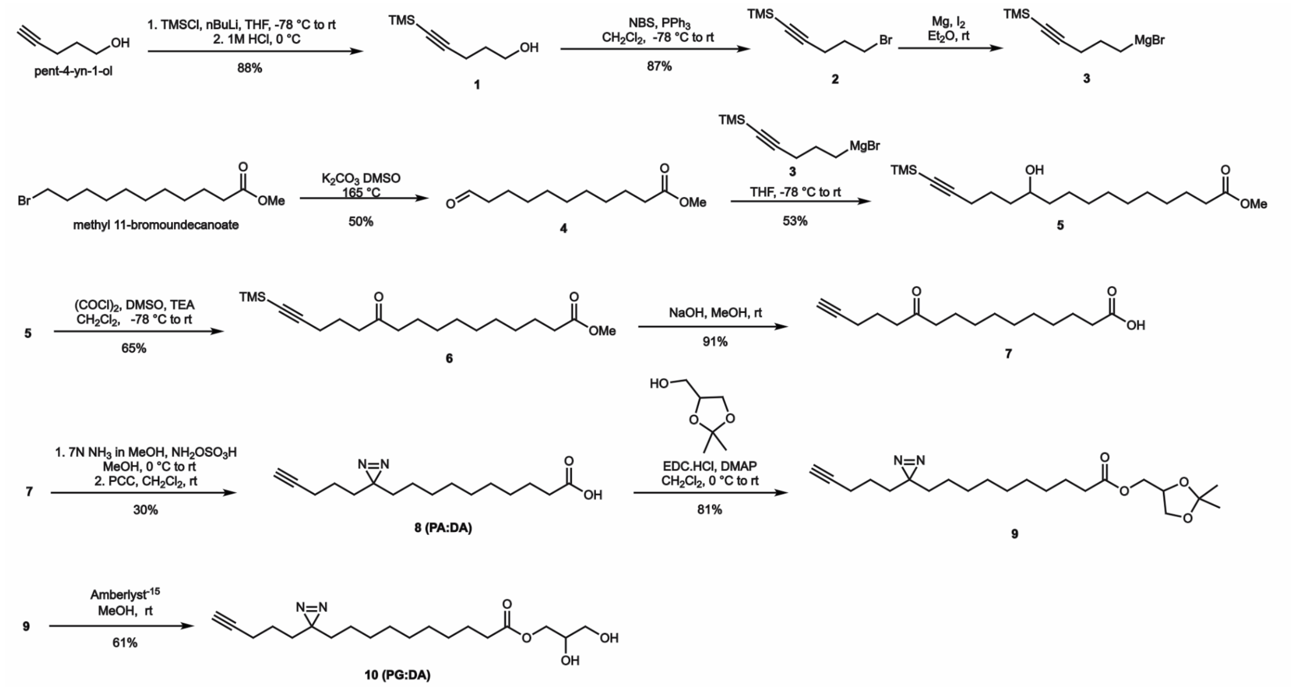

Synthesis: The bifunctional derivative, palmitoyl glycerol diazirine alkyne (PG-DA; compound **10**), features a palmitic acid chain with a terminal alkyne and an internal photoactive diazirine group (i.e., palmitic acid diazirine; PA-DA; compound **8**). A palmitic acid diazirine alkyne (**PA-DA**) was synthesized following previously established procedures with some modifications[13]. The reported synthesis of the aldehyde **4** used a lactone as a starting material, which is quite expensive and not readily commercially available. To address this shortcoming, we used a different approach. We started with an affordable and widely available alkyl halide, and converted it directly into the aldehyde using Kornblum oxidation[14]. Next, we encountered some difficulty with the earlier reported protocol[13] for the Grignard reaction using (5-chloropent-1-yn-1-yl)trimethylsilane. Specifically, a substantial portion of the compound remained unreacted while attempting to prepare the Grignard reagent from the TMS-protected pentynyl chloride. Considering the superior reactivity of a bromide compared to a chloride with magnesium, we decided to prepare (5-bromopent-1-yn-1-yl)trimethylsilane and used this as a substrate for the Grignard reaction. First, TMS protection of the alkyne of pent-4-yn-1-ol was carried out to give **1** in 88% yield. An Appel-type reaction[15] was used to convert the alcohol into the bromide **2** (87% yield). Treatment of a freshly prepared Grignard reagent **3** with aldehyde **4** afforded **5** in 53% yield. Subsequent steps were carried out using established protocols to produce **8** (**PA-DA**). For the synthesis of bifunctional MAG palmitic acid derivative, **PG-DA**, the following sequence of reactions was used. First, **8** (**PA-DA**) was treated with 1,2-isopropylideneglycerol in the presence of 1-ethyl-3-(3-dimethyl-aminopropyl)-carbodiimide hydrochloride (EDC.HCl) and the desired product **9** was isolated in 81% yield. Deprotection of the isopropylidene group of **9** using Amberlyst-15 gave **10** (**PG-DA**) in 69% yield.

General. All chemicals were purchased from Sigma-Aldrich and TCI, unless otherwise mentioned and used as received. All reactions were carried out under an atmosphere of nitrogen (N_2) or argon (Ar). Glassware was oven- or flame-dried prior to use. Analytical thin-layer chromatography (TLC) was performed using Silica Gel 60 F254 pre-coated plates (0.25 mm thickness, Merck), and visualized with staining with potassium permanganate ($KMnO_4$) or phosphomolybdic acid (PMA) solutions. Column chromatography was performed on Rankem silica gel (100-200 mesh). Nuclear magnetic resonance (NMR) spectra were recorded using deuterochloroform ($CDCl_3$), as the solvent. ^1H, ^{13}C spectra were recorded on JEOL 400 MHz or Bruker 400 MHz (or 100 MHz for ^{13}C) NMR spectrometers. The internal standards for the recorded NMR spectra were either residual solvent signals (Chloroform, δ_H = 7.26 ppm, δ_C = 77.2 ppm and or an internal standard tetramethylsilane (δ_H =0.00 ppm, δ_C = 0.00 ppm). Chemical shifts (δ) are reported in ppm and coupling constants (J) in Hz, multiplicities were reported by the following abbreviations: s (singlet), broad singlet (bs), d (doublet), dd (doublet of doublet), t (triplet), q (quartet), m (multiplet). High-resolution mass spectra were obtained from high-resolution mass spectrometry (HRMS)–electrospray ionization–Q-TOF–LC–MS/MS (Sciex)

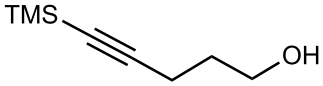

5-(Trimethylsilyl)pent-4-yn-1-ol (1). Compound **1** was synthesized according to a procedure reported previously (Harris et al. 2014) with some modifications[16], and the analytical data collected by us is consistent with literature values. Briefly, in a flame-dried, two-necked round-bottom flask, containing 4-pentyn-1-ol (5.0 g,59.44 mmol, 1.0 equiv.) and anhydrous THF (100 mL) were added, and the mixture was cooled to -78 °C. To this, *n*-butyllithium (52 mL, 2.5 M in hexanes, 130.76 mmol, 2.2 equiv.) was added dropwise over a period of 30 min and the temperature was maintained at -78 °C. Next, chlorotrimethylsilane (22.63 mL, 178.32 mmol, 3.0 equiv.) was added dropwise, and the reaction mixture was gradually warmed to room temperature and stirred for an additional 16 h. The reaction mixture was subsequently cooled to 0 °C and acidified with 1 M HCl (150 mL), followed by stirring for 1 h. The resulting mixture was then extracted with Et_2O(3 x 100 mL), and the combined organic layer was washed with water (250 mL), sat. $NaHCO_3$ (200 mL), and brine (200 mL), dried over Na_2SO_4, and concentrated *in vacuo*. The obtained residue was purified by silica gel column chromatography (elution with gradient 10–25% EtOAc/ hexanes) to afford the compound **1** as a colourless oil (8.17 g, 88%). **TLC** R_f = 0.2 (10% EtOAc/ Hexanes; TLC stain, $KMnO_4$); **^{1}H NMR** (400 MHz, $CDCl_3$): δ 3.75 (t, J = 6.1 Hz, 2H), 2.34 (t, J = 6.9 Hz, 2H), 1.76 (quint, J = 6.6 Hz, 2H), 1.72 (bs, 1H), 0.14 (s, 9H); **HRMS** (*m/z*): [M + H]+ calcd**.** for $C_8H_{17}OSi$, 157.1048; found, 157.1051.

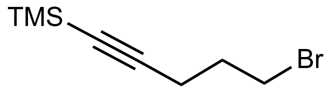

(5-Bromopent-1-yn-1-yl)trimethylsilane (2). Compound **2** was synthesized according to a procedure reported previously (Yao et al. 2002) with some modifications[17], and the analytical data collected by us is consistent with literature values. To a solution of compound **1** (5.1 g, 32.63 mmol, 1 equiv.) in anhydrous CH_2Cl_2 (100 mL). Triphenylphosphine (10.27 g, 39.16 mmol, 1.2 equiv.) was added portion wise at –78 ºC under N_2 atmosphere. *N*-Bromosuccinimide (6.39 g, 35.89 mmol, 1.1 equiv.) was then slowly added, and the reaction mixture was gradually warmed to room temperature and stirred for 6 h. After completion of reaction (TLC analysis), Et_2O (400 mL) was added to the reaction mixture and washed with sat. $NaHCO_3$ (2 x 200 mL), brine (200 mL), dried over Na_2SO_4, and concentrated *in vacuo*. The resulting crude was purified by silica gel column chromatography (elution with 100% hexanes) to afford the **2** as a colourless oil (6.22 g, 87%). **TLC** R_f = 0.5 (100% hexanes; TLC stain, $KMnO_4$) **^{1}H NMR** (400 MHz, $CDCl_3$): δ 3.51 (t, J = 6.5 Hz, 2H), 2.41 (t, J = 6.8 Hz, 2H), 2.04 (quint, J = 6.6 Hz, 2H), 0.15 (s, 9H).

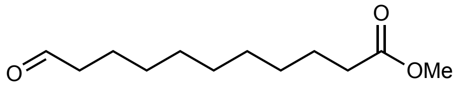

Methyl 11-oxoundecanoate (4). Compound **4** was synthesized according to a procedure reported previously (Ravi, S. et al. 2001) with some modifications[14], and the analytical data collected by us is consistent with literature values. To a solution of methyl 11-bromoundecanoate (0.5 g, 1.791 mmol, 1 equiv.) in anhydrous DMSO (5 mL), $NaHCO_3$ (0.33 g, 3.94 mmol, 2.2 equiv.) was added. The reaction mixture was refluxed at 165 ºC for 15 min and then cooled to room temperature, diluted with ice cold water (10 mL), and extracted with Et_2O (2 x 25 mL). The combined organic layer was washed with water (25 mL), sat. $NaHCO_3$ (20 mL), and brine (20 mL). The resulting organic layer was dried over Na_2SO_4, and concentrated *in vacuo*. The crude was purified by silica gel column chromatography (elution with gradient 10–25% EtOAc/ hexanes) to afford the compound **4** as a colourless oil (0.19 g, 50%). **TLC** R_f = 0.5 (10% EtOAc/ hexane; TLC stain, $KMnO_4$); **^{1}H NMR** (400 MHz, $CDCl_3$): δ 9.76 (t, J = 1.9 Hz, 1H), 3.66 (s, 3H), 2.41(td, J = 77.4, 1.9 Hz, 2H), 2.30 (t, J = 7.5 Hz, 2H), 1.66–1.58 (m, 4H), 1.36–1.25 (m, 10H); **HRMS** (*m/z*): [M + H]+ calcd. for $C_{12}H_{23}O_3$, 215.1647; found, 215.1636.

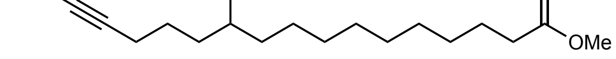

Methyl 11-hydroxy-16-(trimethylsilyl)hexadec-15-ynoate (5). <u>*Preparation of Grignard reagent* (**3**)</u>*:* The Grignard reagent was prepared based on the procedure reported by (Harris et al. 2014) with modifications[16]. Reaction flasks, glass syringes, needles and magnetic stir bars were dried at 110 °C in an oven for 2 h. Magnesium turnings were treated with a 10% aq. HCl solution, followed by subsequent rinsing with water and acetone. The magnesium turnings were then dried in the

oven at 110 ºC for 2 h. Before commencement of the reaction, all items in the oven were gradually cooled to room temperature in a desiccator. The solvents used for the reaction Et_2O and tetrahydrofuran (THF) were dried over sodium metal and then freshly distilled. Also freshly distilled bromide **2** (5-10 mm Hg (Torr), 85-90 ºC; 70-80% yield) was used for the preparation for the Grignard reagent. A 100 mL Schlenk round-bottom flask was charged with the magnesium turnings (0.35 g, 14.45 mmol, 1.2 equiv.) and heated with a heat gun for 10 min under vacuum, and then cooled to room temperature under N_2 atmosphere. A magnetic stir bar was introduced into the flask, and anhydrous Et_2O (16 mL) was added, followed by the addition of a catalytic amount of iodine (8.6 mg, 0.068 mmol, 0.006 equiv.). The mixture was stirred at room temperature for 10 min. A solution of **2** (2.64 g, 12.04 mmol, 1.0 equiv.) in Et_2O (16 mL) was prepared and a small portion, approximately 20%, was added dropwise, and then reaction mixture was stirred at room temperature for 10-15 min until the colour of the solution transitioned from brown/red to colorless, signifying the initiation of the Grignard reagent. The remaining solution of **2** was then added dropwise over 15 min and the reaction mixture was stirred at room temperature for another 3 h. The Grignard reagent **3** was used immediately for the next step.
Grignard reaction: A solution of methyl 11-oxoundecanoate **4** (2.2 g, 10.27 mmol, 1.0 equiv.) in anhydrous THF (33 mL) was cooled to -78 ºC under N_2 atmosphere. The Grignard solution **3** was added dropwise to the stirring solution over 15 min. The reaction mixture was then gently warmed to 0 ºC and stirred for 1 h. Saturated NH_4Cl solution (250 mL) was used to quench the reaction. The product was extracted with EtOAc (3 x 100 mL), and the combined organic layers was dried with Na_2SO_4, and concentrated in *vacuo*. The resulting crude was purified by silica gel column chromatography (elution with gradient 10–20% EtOAc/ hexanes) to afford the compound **5** as a colorless oil (1.93 g, 53%). **TLC** R_f = 0.4 (20% EtOAc/ hexanes, stain with $KMnO_4$); The analytical data collected by us is consistent with reported literature (Hulce et al. 2013)[13] values: **^{1}H NMR** (400 MHz, $CDCl_3$): δ 3.64 (s, 3H), 3.63–3.55 (m, 1H), 2.28 (t, J = 7.5 Hz, 2H), 2.23 (t, J = 6.5 Hz, 2H), 1.63–1.38 (m, 8H), 1.27 (d, J = 4.6 Hz, 12H), 0.12 (s, 9H); **HRMS** (*m/z*): [M + H]+ calcd. for $C_{20}H_{39}O_3Si$, 355.2668; found, 355.2667.

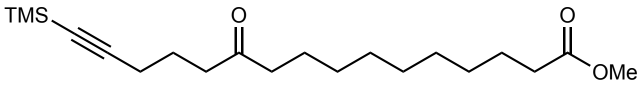

Methyl 11-oxo-16-(trimethylsilyl)hexadec-15-ynoate (6). Compound **6** was synthesized according to a procedure reported previously (Hulce et al. 2013)[1] with some modifications, and the analytical data collected by us is consistent with literature values. *Swern Oxidation:* To a stirring solution of Oxalyl chloride (0.87 mL, 10.16 mmol, 2.0 equiv.) in anhydrous CH_2Cl_2 (65 mL), DMSO (1.45 mL, 20.32 mmol, 4.0 equiv.) was added dropwise at -78 ºC and the reaction mixture was stirred for 30 min. Subsequently, solution of compound **5** (1.8 g, 5.08 mmol, 1.0 equiv) in CH_2Cl_2 (5 mL) was added dropwise, and the reaction mixture was stirred for another 30 min at -78 ºC. Lastly, triethylamine (2.84 mL, 20.32 mmol, 4 equiv.) was added, and after 30 min, the reaction mixture

was gently raised to 0 ºC and stirred at this temperature for 1 h. The reaction mixture was then diluted with H_2O (300 mL) and extracted in CH_2Cl_2 (3 x 100 mL). The combined organic layer was washed with brine (150 mL) and dried over Na_2SO_4 and concentrated in *vacuo*. The crude residue was purified by silica gel column chromatography (elution with gradient 5–10% EtOAc/ hexanes) to afford the compound **6** as a white solid (1.17 g, 65% yield): **TLC** R_f = 0.4 (10% EtOAc/ hexanes; TLC stain, $KMnO_4$); **^{1}H NMR** (400 MHz, $CDCl_3$): δ 3.64 (s, 3H), 2.53 (t, *J* = 7.2 Hz, 2H), 2.40 (t, *J* = 7.4 Hz, 2H), 2.28 (t, *J* = 7.5 Hz, 2H), 2.24 (t, *J* = 6.9 Hz, 2H), 1.75 (quintet, *J* = 7.0 Hz, 2H), 1.64–1.52 (m, 4H), 1.32–1.22 (m, 10H), 0.13 (s, 9H); **HRMS** (*m/z*): [M + H]+ calcd. for $C_{20}H_{37}O_3Si$ [M + H]+ 353.2512, found, 353.2507.

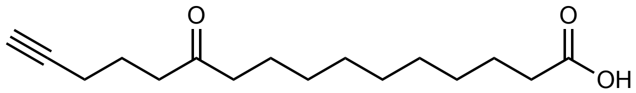

11-Oxohexadec-15-ynoic acid (7). Compound **7** was synthesized according to a procedure reported previously (Hulce et al. 2013) with some modifications[13], and the analytical data collected by us is consistent with literature values. To a solution of **6** (1.16 g, 3.29 mmol, 1.0 equiv.) in MeOH (10 mL), NaOH (0.66 g, 16.45 mmol, 5 equiv.) in H_2O (10 mL) was added dropwise, and the reaction mixture was stirred at room temperature for 16 h. The reaction mixture was acidified to pH 2-3 by treating with 10% aq. HCl at 0 ºC, and extracted with Et_2O (3 x 200 mL). The combined organic layer was washed with brine (250 mL) and dried over Na_2SO_4, and concentrated in vacuo The crude was purified by silica gel column chromatography (elution with gradient 15–25% EtOAc/hexanes, 1% HCO_2H) to afford the compound **7** as a white solid (798 mg, 91%). **TLC** R_f = 0.2 (20% EtOAc/ hexanes with 1% HCO_2H; TLC stain, PMA); **^{1}H NMR** (400 MHz, $CDCl_3$): δ 2.55 (t, *J* = 7.2 Hz, 2H), 2.40 (t, *J* = 7.4 Hz, 2H), 2.34 (t, *J* = 7.5 Hz, 2H), 2.22 (td, *J* = 6.9, 2.8 Hz, 2H), 1.95 (t, *J* = 2.6 Hz, 1H), 1.78 (quintet, *J* = 7.1 Hz, 2H), 1.66–1.52 (m, 4H), 1.34–1.23 (m, 10H); **HRMS** (*m/z*): [M + Na]+ *m/z* calcd. for $C_{16}H_{26}O_3Na$, 289.1780; found 289.1788.

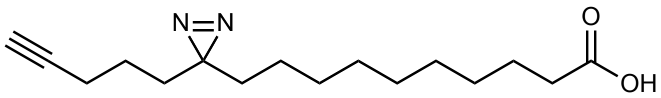

10-(3-(Pent-4-yn-1-yl)-3H-diazirin-3-yl)decanoic acid (PA-DA) (8) Compound **8** (**PA-DA**) was synthesized according to a procedure reported previously (Hulce et al. 2013) with some modifications[13], and the analytical data collected by us is consistent with literature values. The reaction and column chromatography were conducted in the dark due to the light sensitivity of the product. Briefly, in a sealed tube containing a stir bar, 11-oxohexadec-15-ynoic acid (200 mg, 0.75 mmol, 1.0 eq.) was added and cooled to 0 ºC. 7 N NH_3 in MeOH (5.8 mL) was added dropwise and the resulting reaction mixture was stirred for 3 h, while maintaining the temperature at 0 ºC. A solution of hydroxylamine-*O*-sulfonic acid (97 mg, 0.86 mmol, 1.15 equiv.) in MeOH (2.5mL) was gradually added dropwise to the reaction mixture. The seal tube was covered with aluminium foil

and the reaction mixture was stirred at room temperature overnight, and then the solvent was evaporated under a stream of nitrogen gas (N_2). To the remaining residue, Et_2O (10.0 mL) was added, leading to the formation of a suspension containing insoluble salts, which were subsequently filtered off. The filtrate (Et_2O layer) was concentrated under reduced pressure, and the residue obtained was re-dissolved in anhydrous CH_2Cl_2 (12.0 mL) and pyridine (1.75 mL). To this mixture, pyridinium chlorochromate (PCC, 485 mg, 2.25 mmol, 3.0 equiv.) was then added, and the reaction mixture was stirred for 3 h at room temperature. Subsequently, the reaction mixture was passed through a silica pad using a solvent mixture of 70% EtOAc/ hexanes containing 1% formic acid (HCO_2H), and the resulting solution was concentrated *in vacuo*, and the crude was purified by silica gel column chromatography (elution with gradient 10–15% EtOAc/hexanes, 1% HCO_2H) to afford the compound **8** as a white solid (63 mg, 30%). **TLC** R_f = 0.4 (30% EtOAc/hexanes with 1% HCO_2H; TLC stain PMA] **^{1}H NMR** (400 MHz, $CDCl_3$): δ 2.33 (t, J = 7.5 Hz, 2H), 2.15 (td, J = 6.9, 2.6 Hz, 2H), 1.94 (t, J = 2.6 Hz, 1H), 1.61 (quintet, J = 7.5 Hz, 2H), 1.52–1.43 (m, 2H), 1.39–1.15 (m, 14H), 1.12–1.01 (m, 2H); **HRMS** (*m/z*): [M + H]+ calcd. for $C_{16}H_{27}N_2O_2$, 279.2073; found, 279.2071.

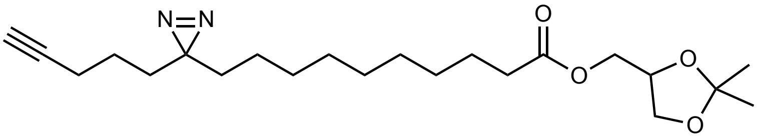

2,2-Dimethyl-1,3-dioxolan-4-yl)methyl 10-(3-(pent-4-yn-1-yl)-3H-diazirin-3-yl)decanoate (9) To a solution of **8** (**PA-DA**, 48 mg, 0.17 mmol, 1.0 equiv.) and 1,2-isopropylideneglycerol (23 mg, 0.17 mmol, 1.0 equiv.) in anhydrous CH_2Cl_2 (4.8 mL), 4-dimethylaminopyridine (5 mg, 0.04 mmol, 0.25 equiv.) was added. The mixture was cooled to 0 ºC and EDC.HCl (0.05 g, 0.26 mmol, 1.5 equiv.) was added in a single portion. The reaction mixture was gradually warmed to room temperature and stirred for 16 h. After completion of reaction (TLC analysis), the reaction was diluted with CH_2Cl_2 (50 mL) and washed with sat. $NaHCO_3$ (2 x 20 mL) and brine (50 mL). The combined organic layer was dried over Na_2SO_4, and concentrated *in vacuo*. The crude residue was purified by silica gel column chromatography (elution with gradient 5–10% EtOAc/ hexanes) to afford **9** as a colorless oil (55 mg, 81%). **TLC** R_f = 0.3 (10% EtOAc/ hexanes; TLC stain, PMA); **^{1}H NMR** (400 MHz, $CDCl_3$): δ 4.35–4.27 (m, 1H), 4.16 (dd, J = 11.5, 4.7 Hz, 1H), 4.12–4.04 (m, 2H), 3.73 (dd, J = 8.4, 6.2 Hz, 1H), 2.33 (t, J = 7.5 Hz, 2H), 2.16 (td, J = 6.9, 2.6 Hz, 2H), 1.94 (t, J = 2.7 Hz, 1H), 1.65–1.56 (m, 2H), 1.51–1.45 (m, 2H), 1.43 (s, 3H), 1.38–1.18 (m, 17H), 1.12–1.01 (m, 2H); **^{13}C NMR** (100 MHz, $CDCl_3$): δ 173.8, 110.0, 83.6, 73.8, 69.0, 66.5, 64.7, 34.2, 33.0, 32.0, 29.4, 29.4, 29.3, 29.3, 29.2, 28.6, 26.8, 25.5, 25.0, 23.9, 22.9, 18.1; **HRMS** (*m/z*): [M + H]+ calcd. for $C_{22}H_{37}N_2O_4$, 393.2753; found, 393.2752.

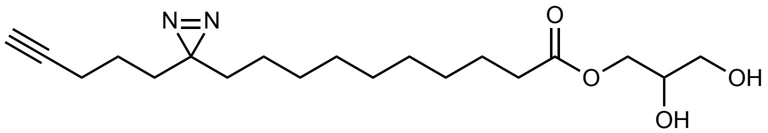

2,3-Dihydroxypropyl 10-(3-(pent-4-yn-1-yl)-3H-diazirin-3-yl)decanoate (PG-DA) (10). Amberlyst-15 (H^+ form, 88 mg, 0.28 mmol, 2 equiv.) was added to a solution of **compound 9** (55 mg, 0.140 mmol, 1 equiv.) in MeOH (9.0 mL). The resulting reaction mixture was stirred for 18 h at room temperature. After completion of reaction (TLC analysis), Amberlyst-15 was filtered off, and the filtrate was concentrated *in vacuo*. The crude residue was purified by silica gel column chromatography (elution with gradient 30–60% EtOAc/ hexanes) to afford the desired compound **10 (PG-DA)** as a colorless oil (30 mg, 61%). **TLC** R_f = 0.5 (60% EtOAc/hexanes; TLC stain, PMA); **^{1}H NMR** (400 MHz, $CDCl_3$): δ 4.21–4.07 (m, 2H), 3.95–3.87 (m, 1H), 3.68 (dd, J = 11.5, 3.9 Hz, 1H), 3.58 (dd, J = 11.5, 5.9 Hz, 1H), 2.91 (bs, 1H), 2.56 (bs, 1H), 2.33 (t, J = 7.6 Hz, 2H), 2.14 (td, J = 7.0, 2.6 Hz, 2H), 1.94 (t, J = 2.7 Hz, 1H), 1.67–1.54 (m, 2H), 1.51–1.42 (m, 2H), 1.38–1.15 (m, 14H), 1.11–1.00 (m, 2H).;**^{13}C NMR** (100 MHz, $CDCl_3$) δ 174.5, 83.6, 70.4, 69.0, 65.2, 63.5, 34.2, 32.9, 31.9, 29.4, 29.3, 29.2, 29.2, 29.2, 28.6, 25.0, 23.9, 22.9, 18.1; **HRMS** *(m/z):* [M + H]+ calcd. for $C_{19}H_{33}N_2O_4$, 353.2440; found 353.2437.

^{1}H NMR of 5-(Trimethylsilyl)pent-4-yn-1-ol (1)

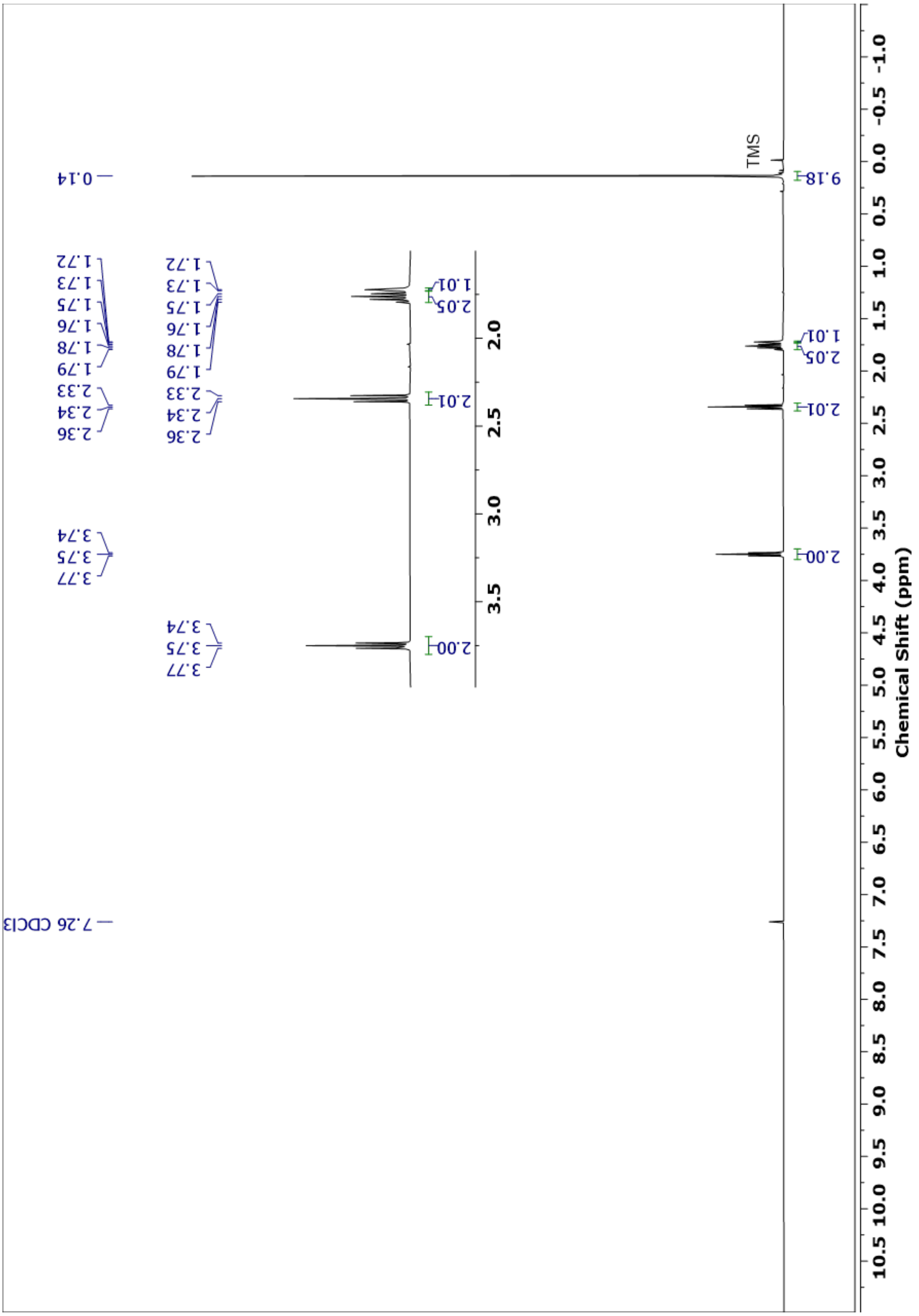

^{1}H NMR of (5-Bromopent-1-yn-1-yl)trimethylsilane (2)

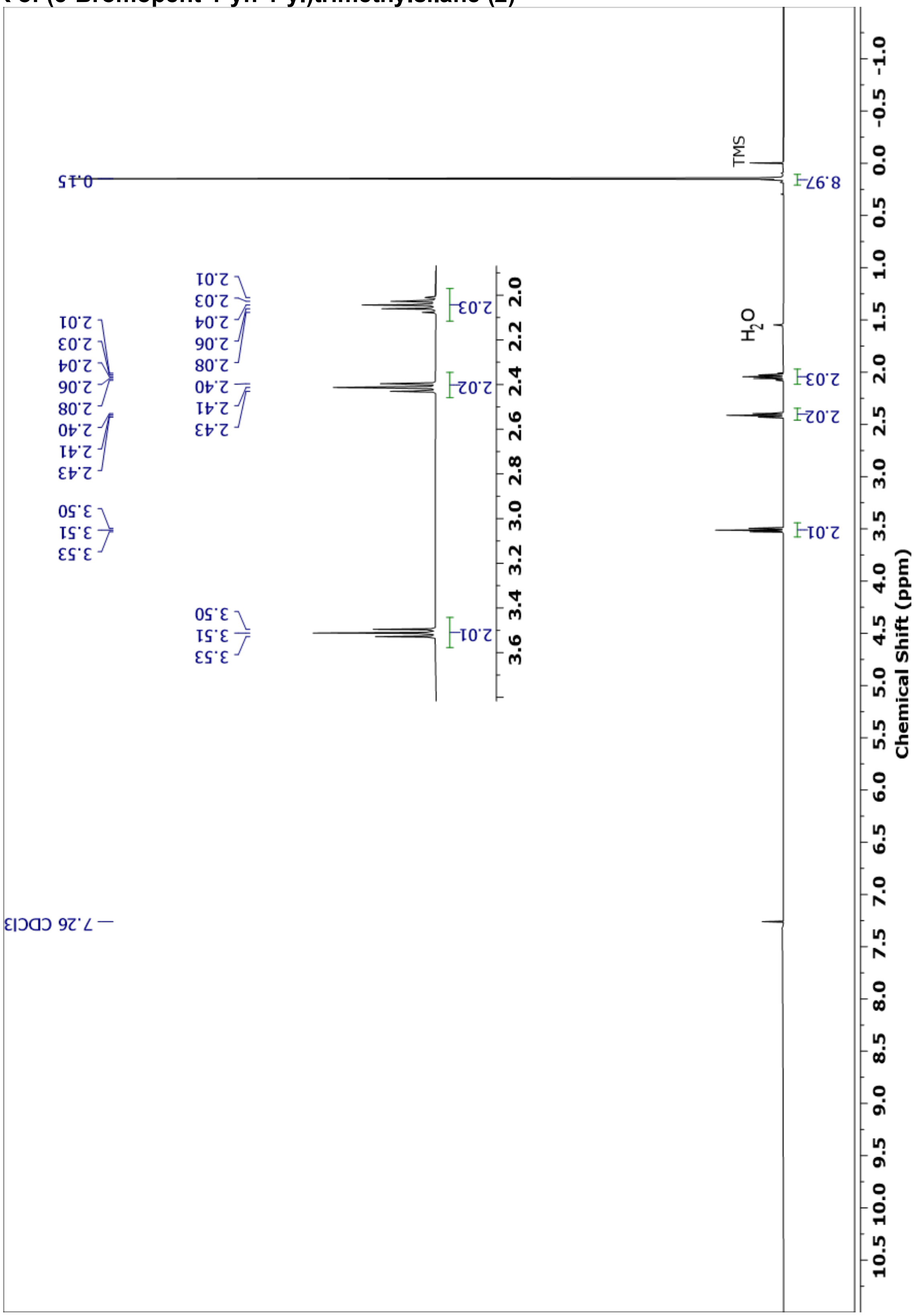

^{1}H NMR of Methyl 11-oxoundecanoate (4)

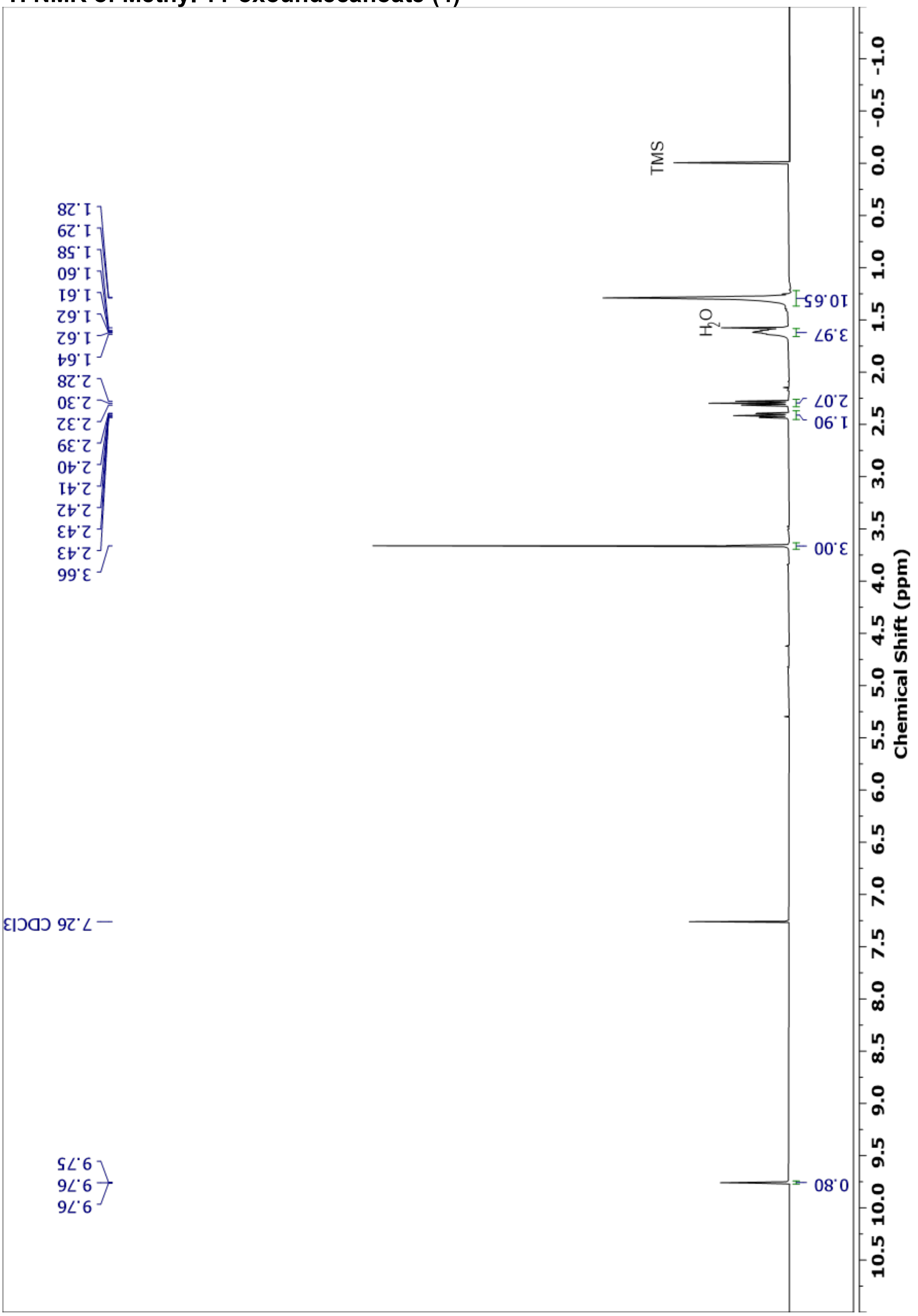

^{1}H NMR of Methyl 11-hydroxy-16-(trimethylsilyl)hexadec-15-ynoate (5)

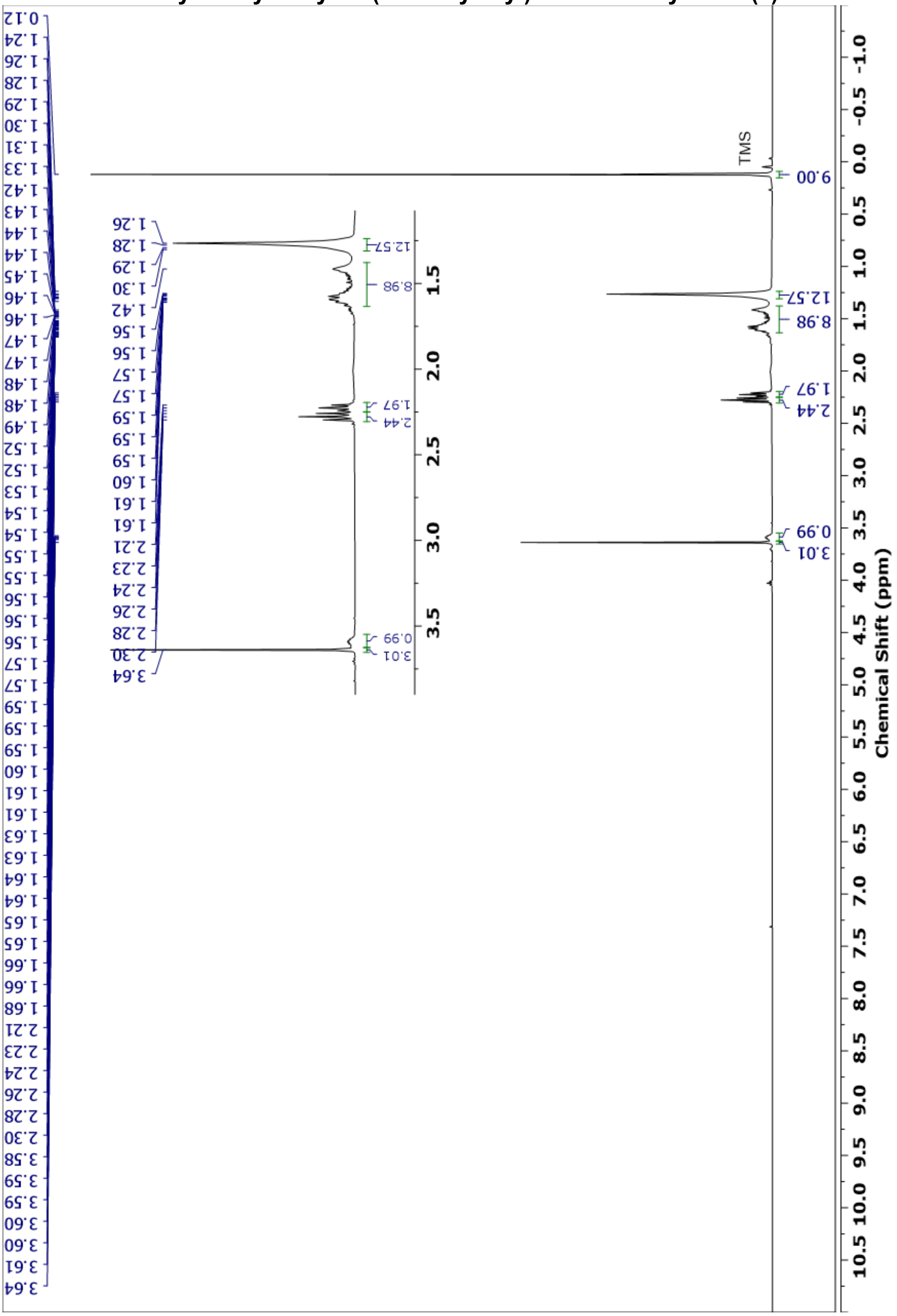

¹H NMR of methyl 11-oxo-16-(trimethylsilyl)hexadec-15-ynoate (6)

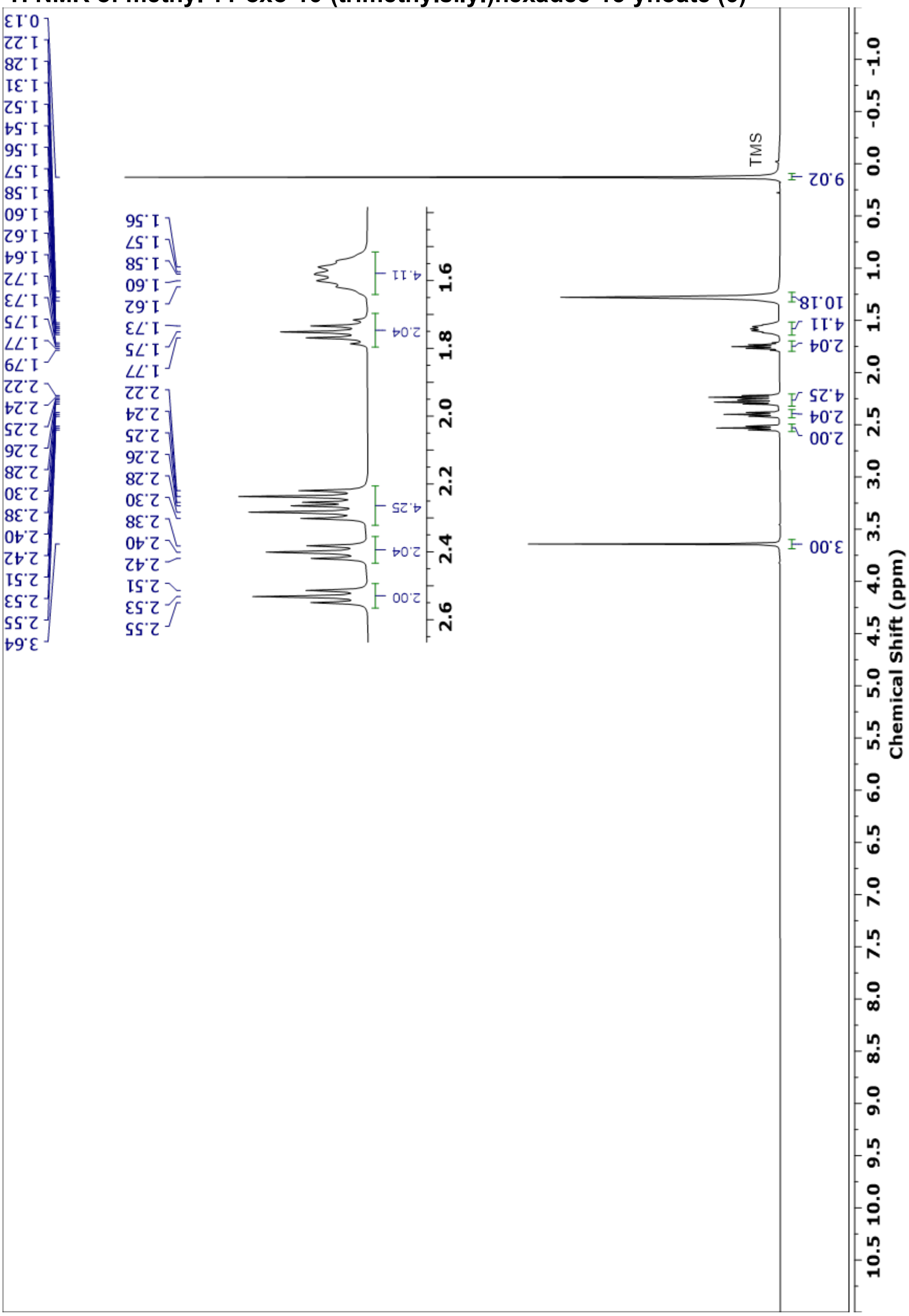

^{1}H NMR of 11-Oxohexadec-15-ynoic acid (7)

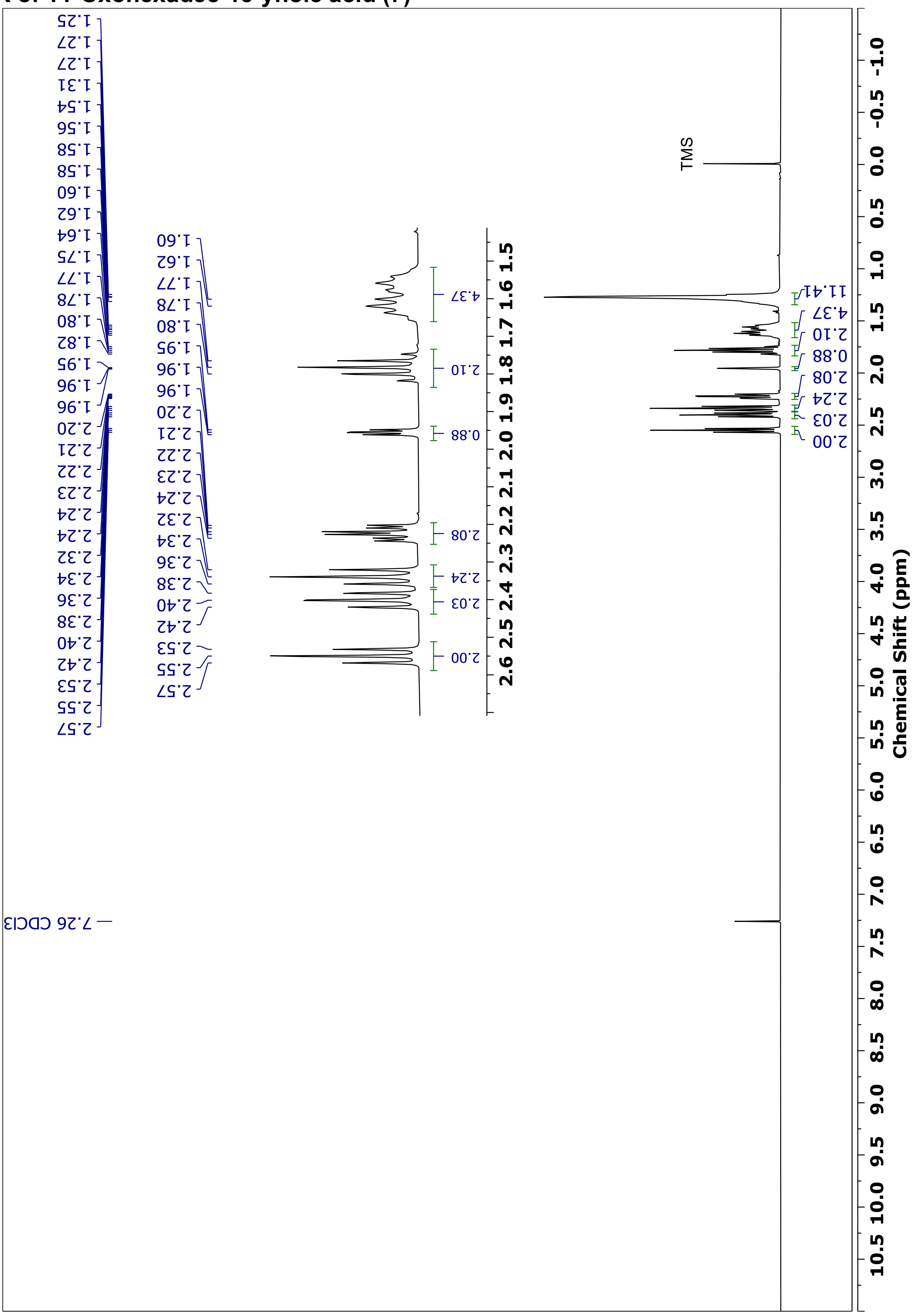

^{13}C NMR of 11-Oxohexadec-15-ynoic acid (7)

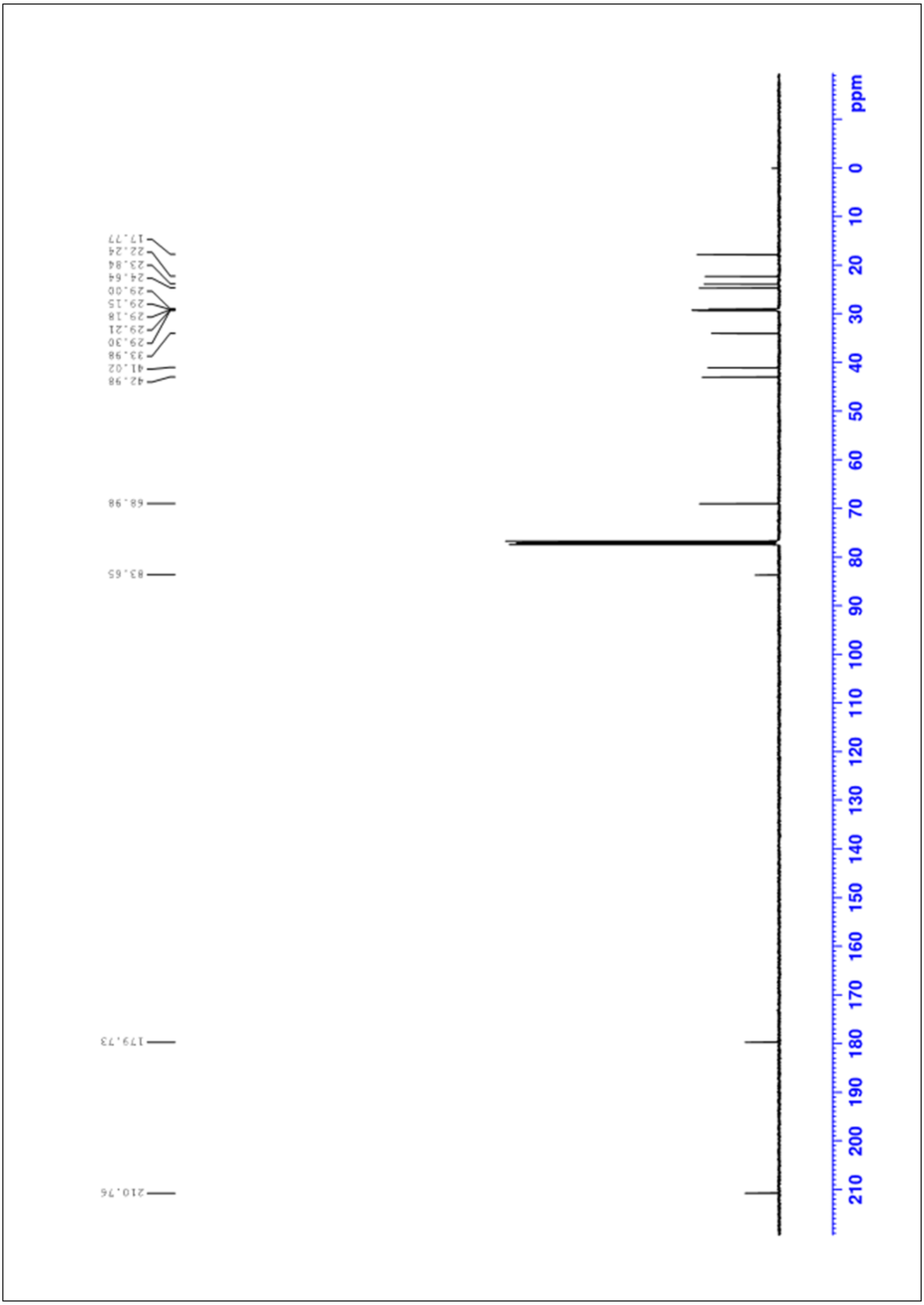

^{1}H NMR of 10-(3-(pent-4-yn-1-yl)-3*H*-diazirin-3-yl)decanoic acid (8) (PA:DA)

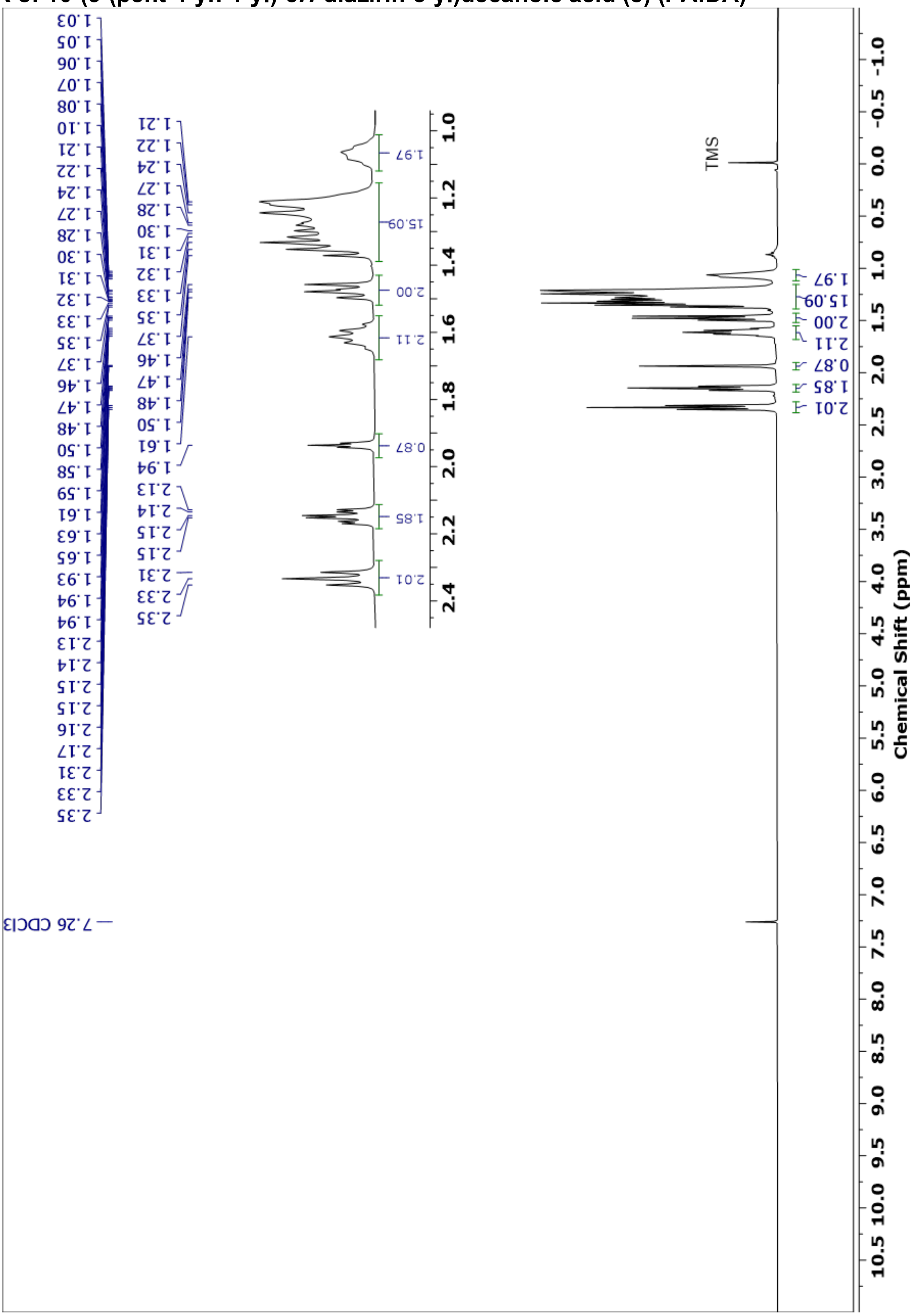

^{13}C NMR of 10-(3-(pent-4-yn-1-yl)-3*H*-diazirin-3-yl)decanoic acid (8) (PA:DA)

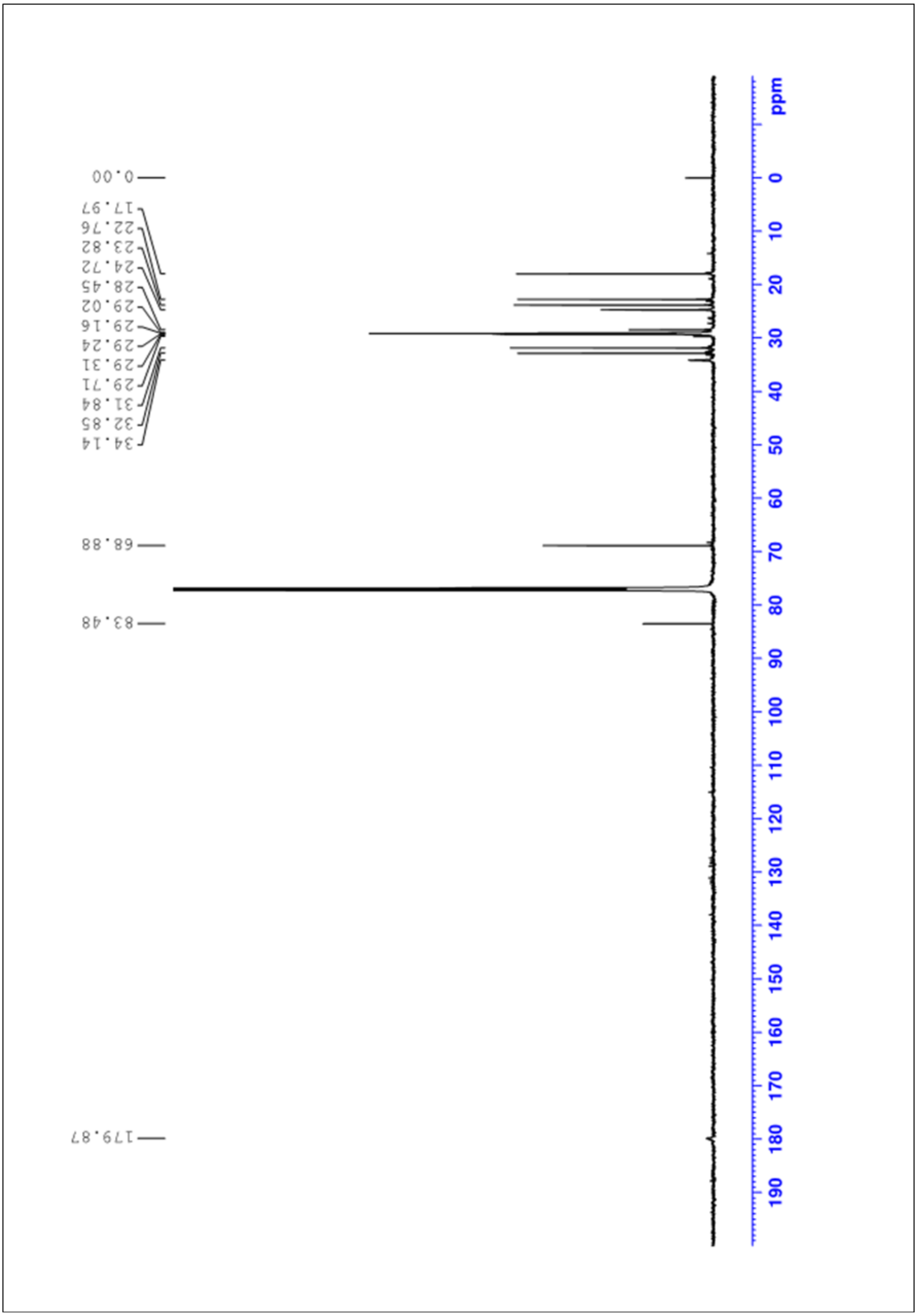

^{1}H NMR of (2,2-Dimethyl-1,3-dioxolan-4-yl)methyl 10-(3-(pent-4-yn-1-yl)-3*H*-diazirin-3-yl)decanoate (9)

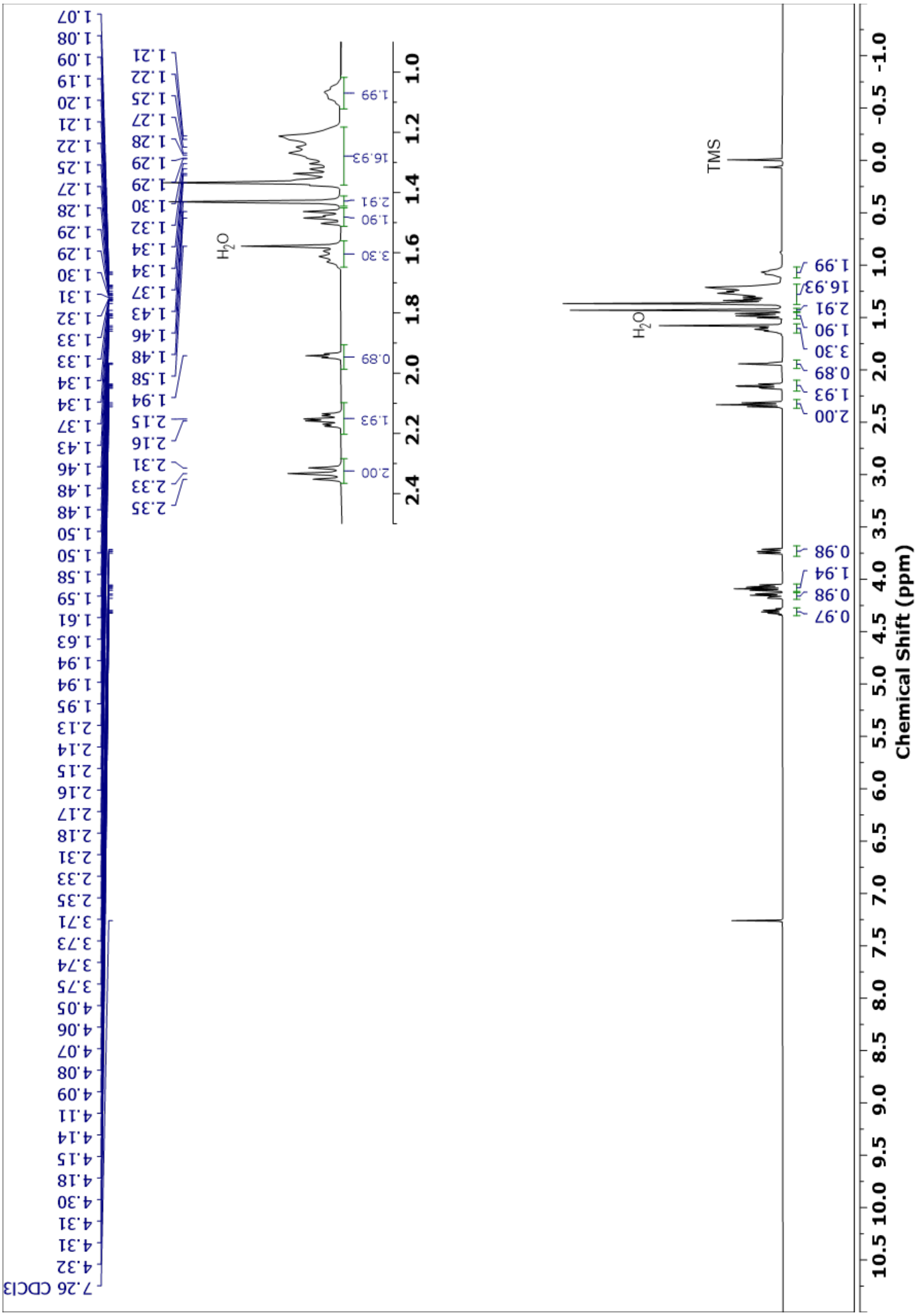

^{13}C NMR of (2,2-Dimethyl-1,3-dioxolan-4-yl)methyl 10-(3-(pent-4-yn-1-yl)-3*H*-diazirin-3-yl)decanoate (9)

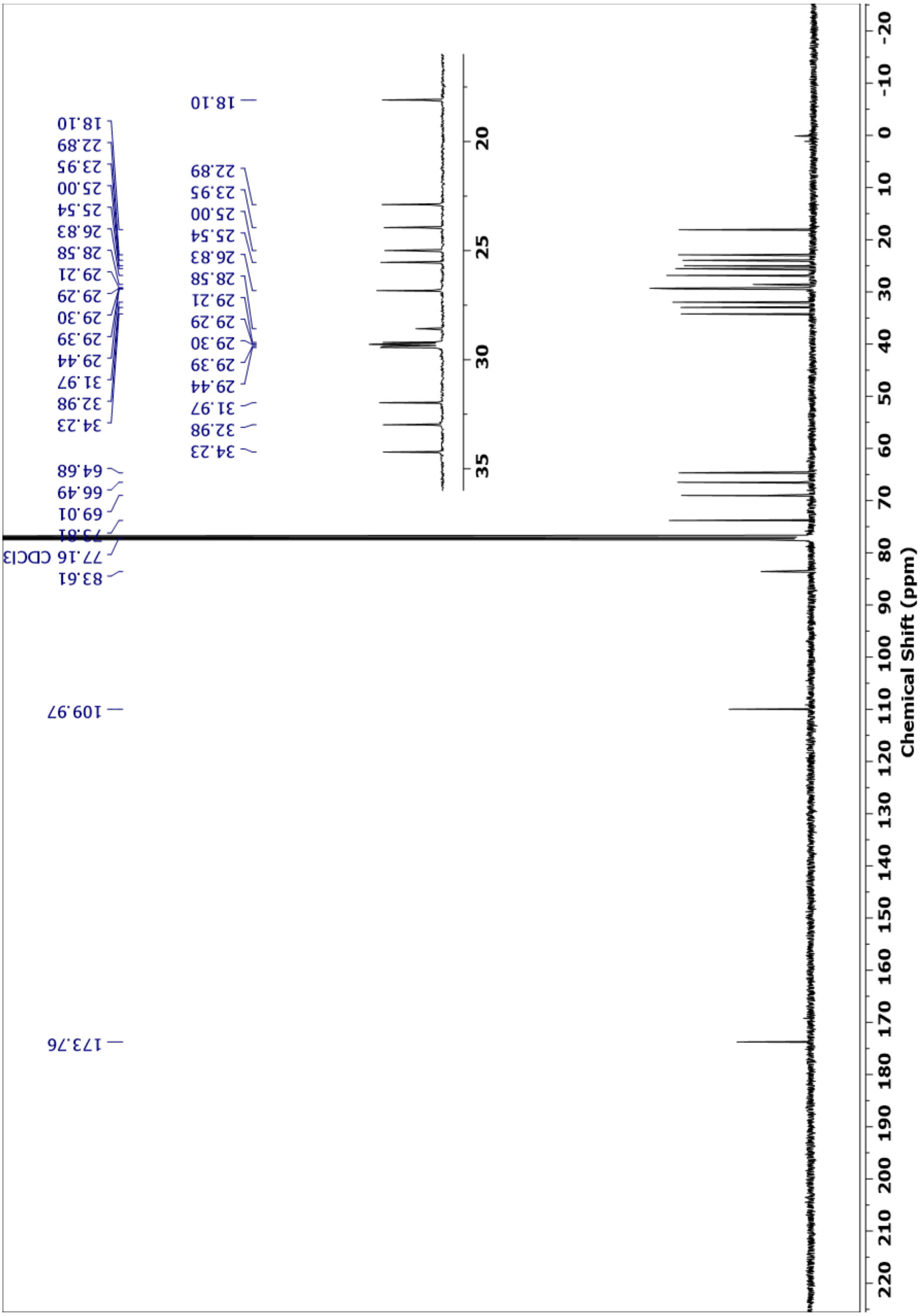

^{1}H NMR of 2,3-dihydroxypropyl 10-(3-(pent-4-yn-1-yl)-3*H*-diazirin-3-yl)decanoate (10) (PG-DA)

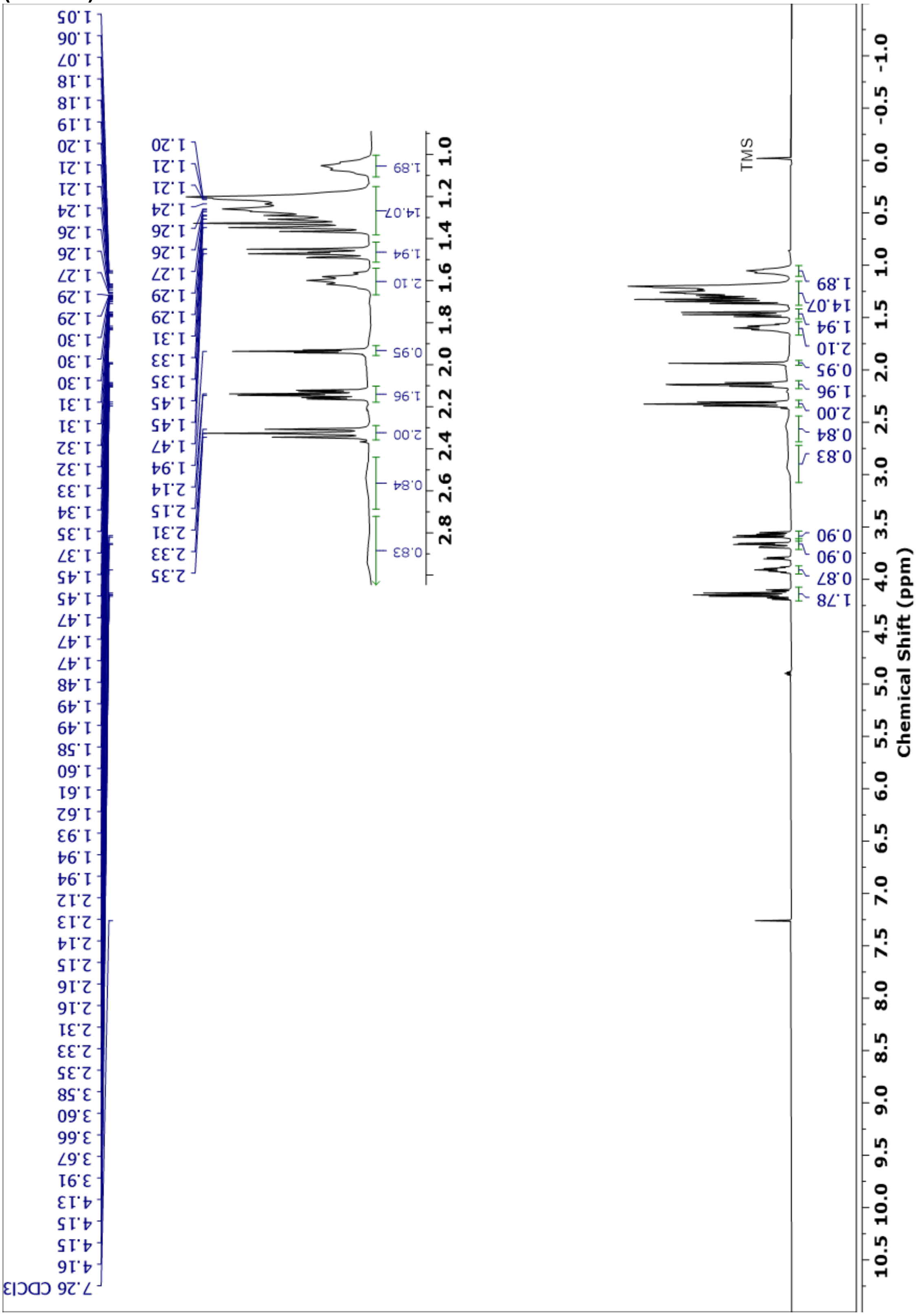

^{13}C NMR of 2,3-dihydroxypropyl 10-(3-(pent-4-yn-1-yl)-3*H*-diazirin-3-yl)decanoate (10) (PG-DA)

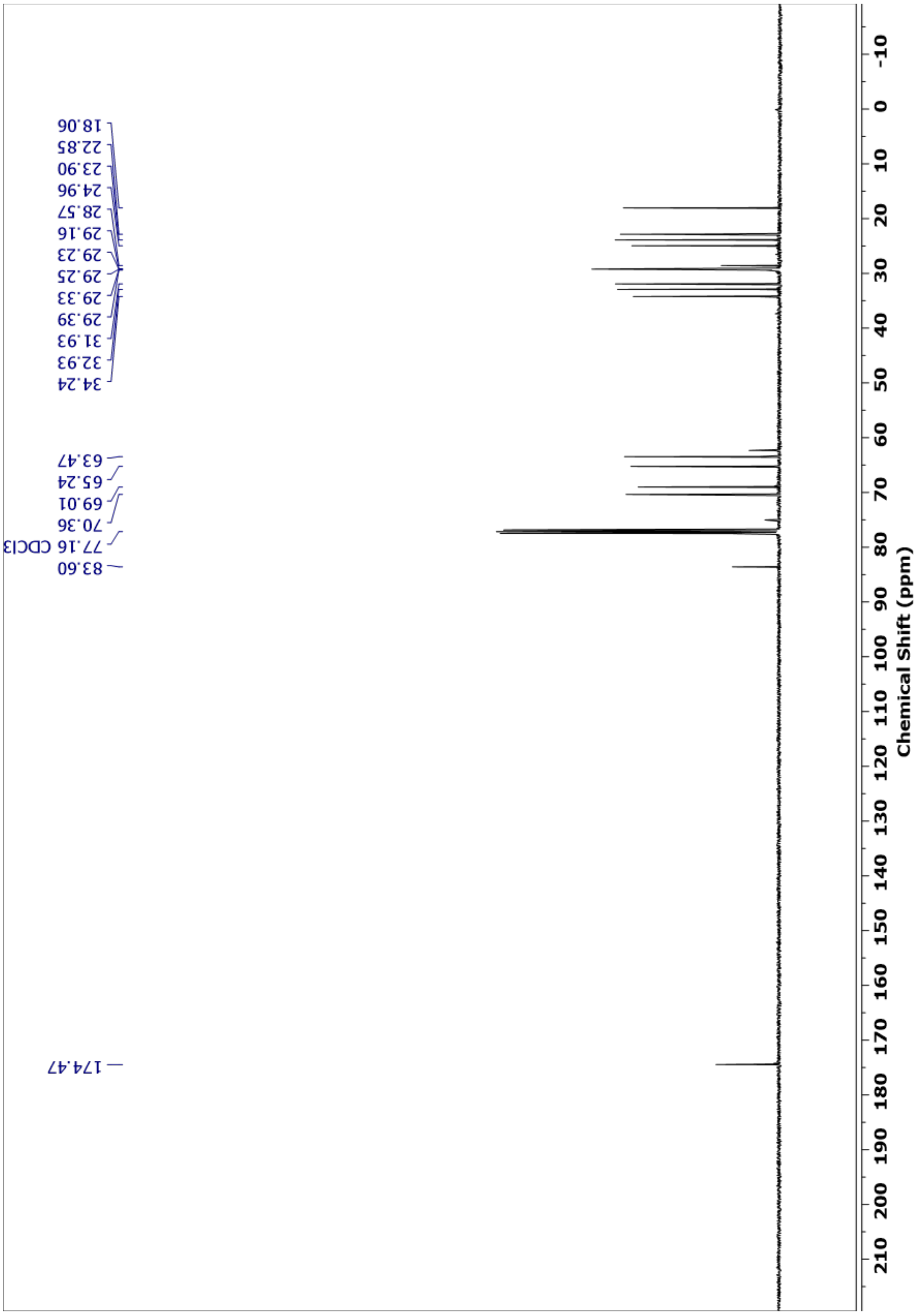

SUPPLEMENTARY REFERENCES.

1 Kumar, K., Pazare, M., Ratnaparkhi, G. S. & Kamat, S. S. CG17192 is a Phospholipase That Regulates Signaling Lipids in the Gut upon Infection. *Biochemistry* **63**, 3000-3010, doi:10.1021/acs.biochem.4c00579 (2024).

2 Kelkar, D. S. *et al.* A chemical-genetic screen identifies ABHD12 as an oxidized-phosphatidylserine lipase. *Nat Chem Biol* **15**, 169-178, doi:10.1038/s41589-018-0195-0 (2019).

3 Chakraborty, A. *et al.* Identification of ABHD6 as a lysophosphatidylserine lipase in the mammalian liver and kidneys. *J Biol Chem* **301**, 108157, doi:10.1016/j.jbc.2025.108157 (2025).

4 Talwadekar, M. *et al.* Metabolic transitions regulate global protein fatty acylation. *J Biol Chem* **300**, 105563, doi:10.1016/j.jbc.2023.105563 (2024).

5 Bateman, A. *et al.* UniProt: the Universal Protein Knowledgebase in 2023. *Nucleic Acids Research* **51**, D523-D531, doi:10.1093/nar/gkac1052 (2023).

6 Thomas, P. D. *et al.* PANTHER: Making genome-scale phylogenetics accessible to all. *Protein Science* **31**, 8-22, doi:10.1002/pro.4218 (2022).

7 Mi, H., Muruganujan, A. & Thomas, P. D. PANTHER in 2013: modeling the evolution of gene function, and other gene attributes, in the context of phylogenetic trees. *Nucleic Acids Res* **41**, D377-386, doi:10.1093/nar/gks1118 (2013).

8 Tan, K. P., Nguyen, T. B., Patel, S., Varadarajan, R. & Madhusudhan, M. S. Depth: a web server to compute depth, cavity sizes, detect potential small-molecule ligand-binding cavities and predict the pKa of ionizable residues in proteins. *Nucleic Acids Res* **41**, W314-321, doi:10.1093/nar/gkt503 (2013).

9 Xu, Y. *et al.* CavityPlus: a web server for protein cavity detection with pharmacophore modelling, allosteric site identification and covalent ligand binding ability prediction. *Nucleic Acids Res* **46**, W374-W379, doi:10.1093/nar/gky380 (2018).

10 Honorato, R. V. *et al.* The HADDOCK2.4 web server for integrative modeling of biomolecular complexes. *Nature protocols*, doi:10.1038/s41596-024-01011-0 (2024).

11 Liu, Y., Patricelli, M. P. & Cravatt, B. F. Activity-based protein profiling: the serine hydrolases. *Proc Natl Acad Sci U S A* **96**, 14694-14699 (1999).

12 Joshi, A. *et al.* Biochemical characterization of the PHARC-associated serine hydrolase ABHD12 reveals its preference for very-long-chain lipids. *Journal of Biological Chemistry* **293**, 16953-16963, doi:10.1074/jbc.RA118.005640 (2018).

13 Hulce, J. J., Cognetta, A. B., Niphakis, M. J., Tully, S. E. & Cravatt, B. F. Proteome-wide mapping of cholesterol-interacting proteins in mammalian cells. *Nat Methods* **10**, 259-264, doi:10.1038/nmeth.2368 (2013).

14 Ravi, S., Padmanabhan, D. & Mamdapur, V. R. Macrocyclic musk compounds: Synthetic approaches to key intermediates for exaltolide, exaltone and dilactones. *Journal of the Indian Institute of Science* **81**, 299-312 (2001).

15 van Kalkeren, H. A., Leenders, S. H., Hommersom, C. R., Rutjes, F. P. & van Delft, F. L. In situ phosphine oxide reduction: a catalytic Appel reaction. *Chemistry* **17**, 11290-11295, doi:10.1002/chem.201101563 (2011).

16 Harris, M. R., Konev, M. O. & Jarvo, E. R. Enantiospecific intramolecular Heck reactions of secondary benzylic ethers. *J Am Chem Soc* **136**, 7825-7828, doi:10.1021/ja5026485 (2014).

17 Yao, G. & Steliou, K. Synthetic studies toward bioactive cyclic peroxides from the marine sponge Plakortis angulospiculatus. *Org Lett* **4**, 485-488, doi:10.1021/ol016943y (2002).
