# Supplementary material for: Chemoproteomics identifies protein ligands for monoacylglycerol lipids
Source: Commun Chem. 2025 Jul 4;8:197. doi: 10.1038/s42004-025-01589-w (PMC12227648; doi:10.1038/s42004-025-01589-w)
Supplement: Supplementary file 2 — Description of Additional Supplementary files [file 42004_2025_1589_MOESM2_ESM.pdf]

Description of Additional Supplementary files

File name: Supplementary Data 1

Description: A LC-MS/MS based chemoproteomic characterization of the PG-DA probe as a function of UV-crosslinking in different mammalian lysates.

File name: Supplementary Data 2

Description: A LC-MS/MS based chemoproteomic characterization of the PA-DA probe as a function of UV-crosslinking in different mammalian lysates.

File name: Supplementary Data 3

Description: A competitive LC-MS/MS based chemoproteomics experiments comparing the protein ligands of the PG-DA probe versus the PA-DA probe in different mammalian lysates.

File name: Supplementary Data 4

Description: Datasets from the molecular docking of 1-PG into HPCA and TOMM22.
