# Supplementary material for: Chemoproteomics identifies protein ligands for monoacylglycerol lipids
Source: Commun Chem. 2025 Jul 4;8:197. doi: 10.1038/s42004-025-01589-w (PMC12227648; doi:10.1038/s42004-025-01589-w)
Supplement: Supplementary file 7 — Reporting Summary [file 42004_2025_1589_MOESM7_ESM.pdf]

nature portfolio

Corresponding author(s): Siddhesh S. Kamat

Last updated by author(s): Jun 12, 2025

# Reporting Summary

Nature Portfolio wishes to improve the reproducibility of the work that we publish. This form provides structure for consistency and transparency in reporting. For further information on Nature Portfolio policies, see our Editorial Policies and the Editorial Policy Checklist.

## Statistics

For all statistical analyses, confirm that the following items are present in the figure legend, table legend, main text, or Methods section.

| n/a | Confirmed | |
|---|---|---|
| ☐ | ☒ | The exact sample size (*n*) for each experimental group/condition, given as a discrete number and unit of measurement |
| ☐ | ☒ | A statement on whether measurements were taken from distinct samples or whether the same sample was measured repeatedly |
| ☒ | ☐ | The statistical test(s) used AND whether they are one- or two-sided<br>*Only common tests should be described solely by name; describe more complex techniques in the Methods section.* |
| ☒ | ☐ | A description of all covariates tested |
| ☒ | ☐ | A description of any assumptions or corrections, such as tests of normality and adjustment for multiple comparisons |
| ☒ | ☐ | A full description of the statistical parameters including central tendency (e.g. means) or other basic estimates (e.g. regression coefficient) AND variation (e.g. standard deviation) or associated estimates of uncertainty (e.g. confidence intervals) |
| ☒ | ☐ | For null hypothesis testing, the test statistic (e.g. *F*, *t*, *r*) with confidence intervals, effect sizes, degrees of freedom and *P* value noted<br>*Give P values as exact values whenever suitable.* |
| ☒ | ☐ | For Bayesian analysis, information on the choice of priors and Markov chain Monte Carlo settings |
| ☒ | ☐ | For hierarchical and complex designs, identification of the appropriate level for tests and full reporting of outcomes |
| ☒ | ☐ | Estimates of effect sizes (e.g. Cohen's *d*, Pearson's *r*), indicating how they were calculated |

*Our web collection on statistics for biologists contains articles on many of the points above.*

## Software and code

Policy information about availability of computer code

| | |
|---|---|
| Data collection | N/A |
| Data analysis | N/A |

For manuscripts utilizing custom algorithms or software that are central to the research but not yet described in published literature, software must be made available to editors and reviewers. We strongly encourage code deposition in a community repository (e.g. GitHub). See the Nature Portfolio guidelines for submitting code & software for further information.

## Data

Policy information about availability of data

All manuscripts must include a data availability statement. This statement should provide the following information, where applicable:

- Accession codes, unique identifiers, or web links for publicly available datasets
- A description of any restrictions on data availability
- For clinical datasets or third party data, please ensure that the statement adheres to our policy

All data that supports the findings of this study are available in the paper and its associated Supporting Information or are available with the Lead Contact upon reasonable request.
All raw data from the proteomics experiments are available on the PRIDE database with accession numbers: PXD051332 (PG-DA probe ⍰ UV), PXD051775 (PA-DA

probe ⍰ UV), and PXD051399 (PG-DA probe vs PA-DA probe competition).

## Research involving human participants, their data, or biological material

Policy information about studies with human participants or human data. See also policy information about sex, gender (identity/presentation), and sexual orientation and race, ethnicity and racism.

| | |
|---|---|
| Reporting on sex and gender | N/A |
| Reporting on race, ethnicity, or other socially relevant groupings | N/A |
| Population characteristics | N/A |
| Recruitment | N/A |
| Ethics oversight | N/A |

Note that full information on the approval of the study protocol must also be provided in the manuscript.

# Field-specific reporting

Please select the one below that is the best fit for your research. If you are not sure, read the appropriate sections before making your selection.

☒ Life sciences ☐ Behavioural & social sciences ☐ Ecological, evolutionary & environmental sciences

For a reference copy of the document with all sections, see nature.com/documents/nr-reporting-summary-flat.pdf

# Life sciences study design

All studies must disclose on these points even when the disclosure is negative.

| | |
|---|---|
| Sample size | Sample sizes for individual were determined based on literature precedence for similar experiments. |
| Data exclusions | No data was excluded |
| Replication | All attempts at replication were successful |
| Randomization | Not relevant to this study |
| Blinding | Not relevant to this study |

# Reporting for specific materials, systems and methods

We require information from authors about some types of materials, experimental systems and methods used in many studies. Here, indicate whether each material, system or method listed is relevant to your study. If you are not sure if a list item applies to your research, read the appropriate section before selecting a response.

### Materials & experimental systems

| n/a | Involved in the study | |
|---|---|---|
| ☐ | ☒ | Antibodies |
| ☐ | ☒ | Eukaryotic cell lines |
| ☒ | ☐ | Palaeontology and archaeology |
| ☐ | ☒ | Animals and other organisms |
| ☒ | ☐ | Clinical data |
| ☒ | ☐ | Dual use research of concern |
| ☒ | ☐ | Plants |

### Methods

| n/a | Involved in the study | |
|---|---|---|
| ☒ | ☐ | ChIP-seq |
| ☒ | ☐ | Flow cytometry |
| ☒ | ☐ | MRI-based neuroimaging |

## Antibodies

| | |
|---|---|
| Antibodies used | The primary antibodies used in this study were the anti-beta-2 adaptin (AP2B1 subunit of the AP2 adaptor complex) Ab (rabbit IgG) (Invitrogen; Catalog No.: PA1-1066) and anti-His Ab (Mouse IgG) (Abcam; Catalog No.: ab18184) antibodies at a dilution of 1:1000. |

| | |
|---|---|
| | The secondary antibodies used in this study were HRP-conjugated anti-rabbit Ab (Goat IgG) (Invitrogen; Catalog No.: 31460) and HRP-conjugated anti-mouse Ab (Goat IgG) (Abcam; Catalog No.: ab6789) at a dilution of 1:10,000. |
| Validation | All antibodies were validated by the manufacturer, and this information is available for various antibodies on the respective manufacturer's website |

## Eukaryotic cell lines

Policy information about cell lines and Sex and Gender in Research

| | |
|---|---|
| Cell line source(s) | All immortalized mammalian cell lines used in this study were purchased from ATCC |
| Authentication | None of the cell lines used were authenticated |
| Mycoplasma contamination | All cells were routinely stained with 4',6-diamidino-2-phenylindole (DAPI) to ensure that they were devoid of any mycoplasma contamination. No mycoplasma contamination was detected in any cell lines. |
| Commonly misidentified lines (See ICLAC register) | None |

## Animals and other research organisms

Policy information about studies involving animals; ARRIVE guidelines recommended for reporting animal research, and Sex and Gender in Research

| | |
|---|---|
| Laboratory animals | Wild type C57/Bl mice were used for obtaining brains |
| Wild animals | N/A |
| Reporting on sex | N/A |
| Field-collected samples | N/A |
| Ethics oversight | All experiments involving mice used in this study were approved by the Institutional Animal Ethics Committee at IISER Pune constituted as per the guidelines provided by the Committee for the Purpose of Control and Supervision of Experiments on Animals, Government of India (Protocol No.: IISER_Pune IAEC/2023_03/02). |

Note that full information on the approval of the study protocol must also be provided in the manuscript.

## Plants

| | |
|---|---|
| Seed stocks | N/A |
| Novel plant genotypes | N/A |
| Authentication | N/A |
